# Current Research on the Control Mechanisms of Cell Survival and Proliferation as Potential Interaction Sites with Pentacyclic Triterpenoids in Ovarian Cancer

**DOI:** 10.3390/ijms262311622

**Published:** 2025-11-30

**Authors:** Arkadiusz Orchel, Jonasz Skrobek, Anna Kaps, Piotr Paduszyński, Ewa Chodurek

**Affiliations:** Department of Biopharmacy, Faculty of Pharmaceutical Sciences in Sosnowiec, Medical University of Silesia in Katowice, 8 Jedności Str., 41-200 Sosnowiec, Poland; aorchel@sum.edu.pl (A.O.); s83467@365.sum.edu.pl (J.S.); ppaduszynski@sum.edu.pl (P.P.); echodurek@sum.edu.pl (E.C.)

**Keywords:** ovarian cancer, pentacyclic triterpenoids, molecular targets/mechanisms, anti-cancer therapy

## Abstract

The treatment of gynecological cancers is challenging because they are often diagnosed at the advanced stages. Furthermore, available chemotherapeutics increasingly imply the development of resistance in cancer patients. This necessitates the search for alternative solutions that could be used in therapy. One of the possibilities to consider is the use of pentacyclic triterpenoids. They are naturally occurring compounds characterized by a wide range of biological activities. They can also be modified to improve their pharmacological properties. Terpenoids based on oleanane, ursane, and lupane skeletons can modulate the numerous processes occurring in both normal and transformed cells. To develop effective anti-cancer therapy, it is essential to understand the processes regulating the progression and suppression of a given type of cancer. For this reason, it is necessary to assess the influence of the tested compounds on cellular processes such as the cell cycle, epithelial–mesenchymal transition, autophagy, and apoptosis. This article summarizes available information on the effects of pentacyclic triterpenoids on the PI3K/AKT/mTOR, MAPK/ERK, NF-κB, JAK/STAT, Notch, HIF-1α, TGF-β, Wnt/β-catenin, Hippo, and Hedgehog signaling pathways in ovarian cancer cells.

## 1. Introduction

Ovarian cancer (OC) remains one of the most lethal gynecological malignancies, posing a major challenge in contemporary oncology. Due to its insidious onset and nonspecific clinical presentation, the disease is frequently diagnosed at advanced stages, significantly limiting the likelihood of curative treatment. The median age at diagnosis is 63 years, with approximately two-thirds of cases occurring in women over the age of 50 [1]. The current standard of care involves cytoreductive surgery combined with platinum-based chemotherapy, typically cisplatin or carboplatin. While more than 80% of patients initially achieve remission, the majority ultimately relapse, often with tumors that acquire resistance to previously effective regimens. This therapeutic limitation has driven intense research efforts aimed at developing targeted therapies that exploit vulnerabilities in critical oncogenic pathways. Among these, poly(ADP-ribose) polymerase inhibitors have emerged as a paradigm-shifting class of agents [2].

Histopathologically, OC is divided into two main types: type I—slow-growing, low-grade tumors with relatively good outcomes; and type II—the predominant form, high-grade tumors with rapid progression, poor differentiation, and aggressive behavior. Molecular and histologic classification further defines five major subtypes: high-grade serous carcinoma, endometrioid, clear cell, mucinous, low-grade serous carcinoma [2].

Despite its usefulness, this classification overlooks the complexity of ovarian tumorigenesis. A key feature is disruption of cell-cycle checkpoints (G1/S, intra-S, G2/M) caused by cyclin/cyclin-dependent kinase (CDK) overexpression and loss of CDK inhibitors: p21, p27. Translationally controlled tumor protein (TCTP), for example, enhances cyclin D1–CDK4 activity and suppresses p21/p27 [3]. Genomic instability is worsened by deletions in cyclin-dependent kinase inhibitor 2A/B (CDKN2A/B) and CCNE1 amplification. Proliferation is further driven by constitutive activation of phosphatidylinositol-3-kinase/AKT/mammalian target of rapamycin (PI3K/AKT/mTOR) and rat sarcoma virus/mitogen-activated protein kinase (Ras/MAPK) signaling, commonly due to PIK3CA or Kristen rat sarcoma viral oncogene homolog (KRAS) mutations and phosphate and tensin homolog (PTEN) loss [4].

A key factor in ovarian cancer progression is epithelial–mesenchymal transition (EMT), regulated by Twist1, Snail, and Zinc finger E-box binding homeobox 1 (ZEB1), which increase cell motility and invasion [5]. Tumor survival is further supported by widespread TP53 mutations (seen in most HGSC) disrupting p53-dependent apoptosis [6], alongside with B-cell CLL/lymphoma 2 (Bcl-2) overexpression, TCTP–p53/myeloid leukemia-1 (Mcl-1) interactions, and impaired caspase activity [7]. Additional genetic and phenotypic changes, including Breast Cancer Gene (BRCA1/2) mutations, further weaken DNA repair and checkpoints [8]. Emerging molecular variants such as Bruton tyrosine kinase (BTK) and Ephrin type-A receptor 5 (EPHA5) promote metabolic reprogramming, drug resistance, and metastasis [9], and cancer stem cells’ (CSCs) persistence contributes to adaptation to therapy and recurrence [10].

Tumor progression depends on vascular endothelial growth factor (VEGF)/vascular endothelial growth factor receptor-2 (VEGFR-2)-driven angiogenesis [3] and extracellular matrix remodeling that enables invasion [11]. Chronic inflammation creates a cytokine-rich environment (interleukin (IL)-6, IL-8, tumor necrosis factor-α (TNF-α)) and activates nuclear factor kappa-light-chain-enhancer of activated B cells (NF-κB) and signal transducer and activator of transcription 3 (STAT3), promoting proliferation, angiogenesis, and immune escape [12]. Inflammation-associated reactive oxygen species (ROS) further damage DNA and mitochondria while enhancing angiogenesis and metastasis [6,13].

In ovarian carcinogenesis, dysregulated signaling pathways are of crucial significance. PI3K/AKT/mTOR activation promotes proliferation, survival, and angiogenesis, while PIK3CA mutations and PTEN loss potentiate therapy resistance [14]. Hyperactive RAS/MAPK/extracellular signal-regulated kinase (ERK) sustains uncontrolled cell divisions [15], and constitutive NF-κB signaling induces pro-inflammatory cytokines, anti-apoptotic effectors, and immune suppression [12,16]. The Janus kinase (JAK)/STAT axis, particularly STAT3, enhances survival signaling and immunosuppressive tumor microenvironment, correlating with poor prognosis [17].

Developmental pathways like Notch and Wnt/β-catenin support CSCs maintenance, therapy resistance and migration. Notch promotes tumor survival, while β-catenin enhances CSC renewal and metastasis [18,19]. Hippo and Hedgehog (Hh) pathways, via Yes-associated protein/transcriptional coactivator with PDZ-binding motif (YAP/TAZ) and Gli transcription factors, disrupt proliferation–apoptosis balance, boosting invasive and stem-like phenotypes [20,21].

Importantly, these signaling modules do not act in isolation. Chronic inflammation and oxidative stress synergistically activate several pathways generating an inter-connected signaling network that underlies tumor aggressiveness. This intricate molecular crosstalk highlights these pathways as compelling targets for next-generation therapeutic strategies in ovarian cancer.

## 2. Pentacyclic Triterpenoids as Anti-Cancer Compounds

**Figure 1:**
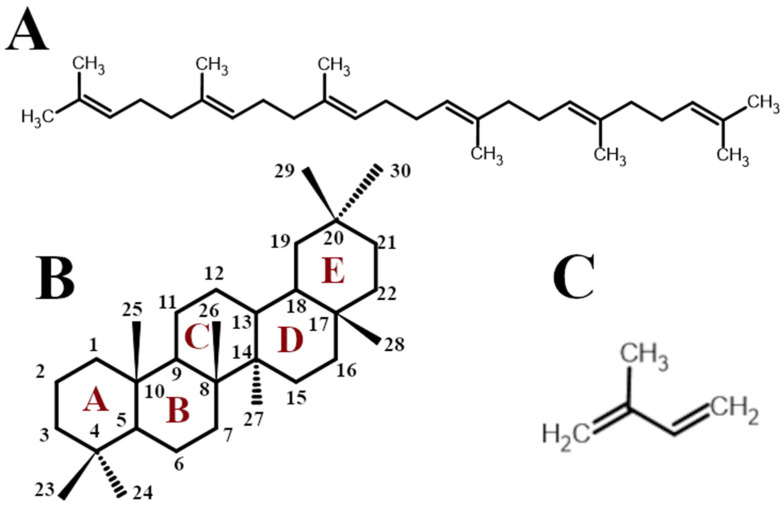
Chemical structure of (**A**) squalene; (**B**) oleanane (the letter numbering of the rings is shown in red); (**C**) isoprene unit.

Terpenes and terpenoids constitute a large group of secondary metabolites comprising more than 50,000 substances. Generally, terpenes are simple hydrocarbons built from five-carbon isoprene (isopentane) units (Figure 1C). Accordingly, their general chemical formula is (C_5_H_8_)n, and the value of n is used to classify them. Triterpenes are composed of 6 isoprene units, totaling 30 carbon atoms, as a terpene unit results from the combination of 2 isoprene units. Terpenoids are formed as a result of chemical modifications of terpene molecules through the introduction of various oxygen-containing structural units, such as hydroxyl, aldehyde, ketone, or carboxyl groups [22,23]. However, numerous authors use the terms terpenes and terpenoids interchangeably without making a clear distinction between them. Squalene (Figure 1A), a simple acyclic triterpene, or its oxygenated derivatives, is a precursor of the large group of polycyclic triterpenoids generated in the cytosol as a result of enzymatic cyclization [24]. The group of triterpenes possessing five fused carbon rings has attracted exceptional interest due to its widespread occurrence in nature and broad spectrum of biological and pharmacological activities. Typical representatives of this group possess molecules composed of five six-carbon rings, or alternatively, four six-carbon rings and one five-carbon ring. The rings are designated in sequence as A, B, C, D, and E (Figure 1B).

Pentacyclic triterpenoids (PTs) occur commonly in nature, and they have been identified in a wide range of organisms such as fungi, terrestrial plants (pteridophytes, monocotyledons, dicotyledons), and marine organisms (i.e., algae, corals, sponges or mollusks) [25]. They are a typical constituent of plant food, including miscellaneous fruits (especially apple or pear peel, strawberries, green pepper), aromatic herbs (oregano, rosemary, and lavender), and pomace olive oil [26,27,28]. PTs can occur in various plant organs, for example, in the case of *Sambucus nigra* shrubs, triterpenes were extracted from leaves, fruits, and bark. Birch bark is an exceptionally rich source of betulin, and to a lesser extent other PTs [27,29]. These compounds are believed to be largely responsible for the therapeutic effects of many popular medicinal herbs, including Centella asiatica (*Centella asiatica* L.; gotu kola) [30], *Lycopus lucidus* [31], Rhododendron arboretum (*Rhododendron arboretum*) [32], Camellia sinensis (*Camellia sinensis* (L.) Kuntze; green tea) [33], Taraxacum officinale (*Taraxacum officinale* F.H. Wigg;. dandelion) [25], Crataegus monogyna (*Crataegus monogyna* Jacq.; hawthorn), Olea europaea (*Olea europaea* L.; olive), Salvia officinalis (*Salvia officinalis* L.; sage) [34], Arctostaphylos uva-ursi (*Arctostaphylos uva-ursi* (L.) Spreng.; bearberry) [27], and Rosa spp. [35]. PTs have been found in roots of Withania somnifera (*Withania somnifera* (L.) Dunal; ashwagandha), Glycyrrhiza glabra (*Glycyrrhiza glabra* L.; licorice), and Astragalus membranaceus (*Astragalus membranaceus* (Fisch.) Bunge; huang qi) [25]. Mediterranean diet ensures relatively high consumption of PTs reaching 400 mg/day. Typical PT intake in Western countries is estimated to be approximately 250 mg/day. Unfortunately, PTs usually have low oral bioavailability, but it can be significantly improved by some factors as a fat-rich meal or an appropriate nanocarrier system [28].

**Figure 2:**
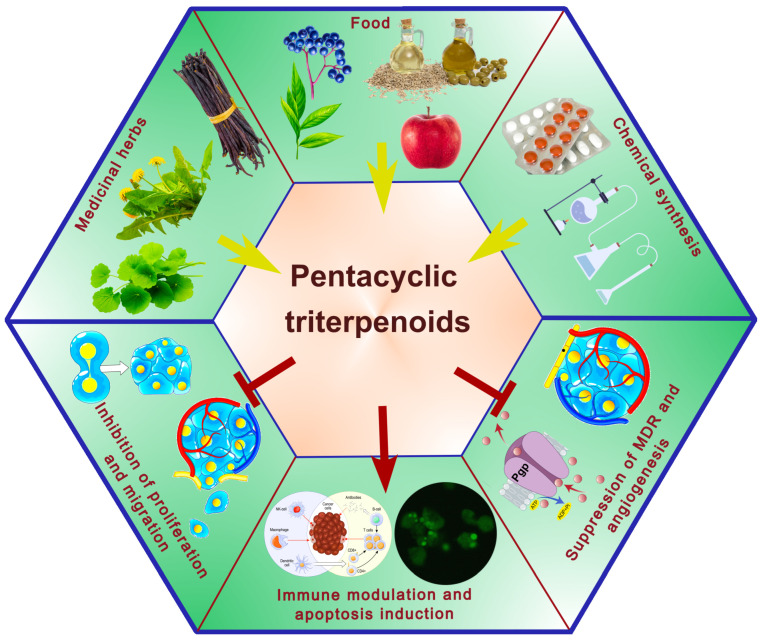
Summary of anti-cancer effects effects (red arrow indicate modulation and induction while red lines ending with perpendicular bars represent an inhibition) of PTs and their main sources (yellow arrows).

Pentacyclic triterpenes demonstrate a broad spectrum of biological activities. In the context of oncological chemoprevention, their anti-inflammatory and antioxidant properties seem to be of particular significance, as inflammation and elevated ROS levels are associated with cancer development and progression. The anti-inflammatory activity has been demonstrated for a large group of PTs, including betulin, lupeol, and acids: ursolic, oleanolic, betulinic, glycyrrhetinic, and boswellic [36,37]. These compounds often inhibit cyclooxygenase (COX) and lipooxygenase (LOX) activity and reduce the release of inflammatory mediators (e.g., TNF-α, IL-1β, IL-6, IL-8). Some PTs have been studied as antioxidative agents, revealing their ability to protect cells against oxidative stress, as well as to prevent lipid peroxidation and oxidative DNA damage. This is achieved through activation of antioxidative enzymes (e.g., superoxide dismutase, catalase) or normalization of the glutathione status [38]. Although the aforementioned processes may contribute significantly to disease chemoprevention, the principal mechanism underlying the anti-cancer effects of PTs consists of their direct actions on tumor cells, including the inhibition of proliferation, induction of apoptosis and cellular differentiation, as well as the suppression of drug resistance, cell migration, and angiogenesis (Figure 2).

## 3. Main Types of Pentacyclic Triterpenoids and Their Effects on Ovarian Cancer Cells

**Figure 3:**
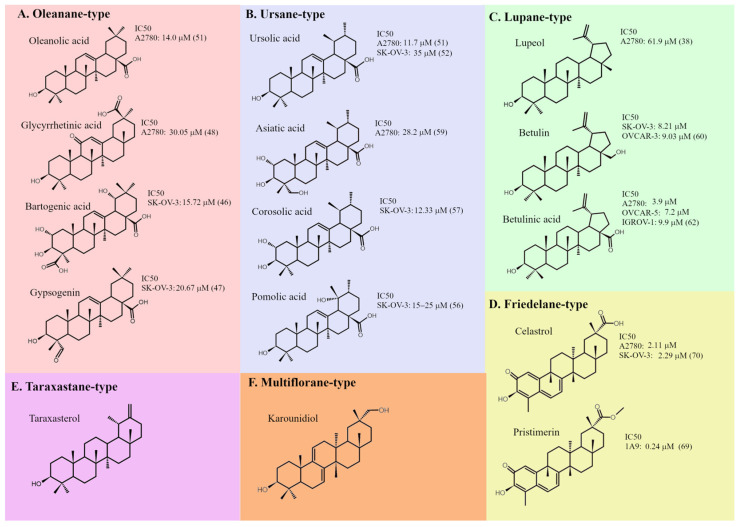
Structures of key representatives of PTs with half maximal inhibitory concentration (IC_50_) values.

Minor structural variations at the molecular level have been used to distinguish several types of PTs, among which the oleanane-, ursane- and lupane-type skeletons are considered most important (Figure 3) [24].

**Figure 4:**
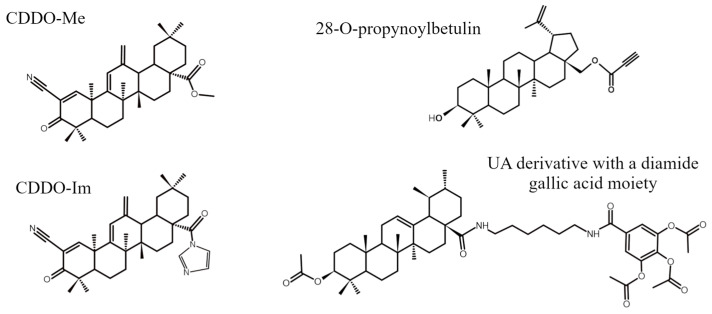
Selected structures of synthetic PTs.

Oleanane-type triterpenoids represent the most abundant group of PTs characterized by molecules built from five six-carbon rings possessing trans conformation with the exception of D-E rings with a cis arrangement [24]. A typical representative of this group, oleanolic acid (OA; C30H48O3), has been identified in thousands of plant species, with *Olea europaea* being the best known [27]. Its precise chemical structure is shown in Figure 3A. It has been demonstrated that OA exhibits moderate anti-proliferative activity against several ovarian cancer cell lines: A2780, SK-OV-3, OVCA420, and CAOV3. Some authors observed an inhibitory effect of OA on cell migration and invasion, as well as its ability to suppress glycolysis and induce autophagy [39,40,41,42]. Cytotoxicity of OA was substantially augmented through various chemical modifications. Siewert et al. [39] have found that even slight differences in structure of OA derivatives result in large variation of their cytotoxic activity and mode of action. They discovered that methyl 3-acetoxy-12-hydroxy-11-oxo-olean-D-12,13-en-28-oate was particularly effective in suppressing the growth of A2780 cells and efficiently induced apoptosis. This acetylated methyl ester had additionally a carbonyl group at C11 and hydroxyl group at C12. Such modifications led to a 70% reduction in the IC_50_ value compared to the parent compound. Among numerous derivatives of oleanolic acid, bardoxolone methyl (BM, methyl-2-cyano-3,12-dioxooleana-1,9(11)-dien-28-oate), also known as CDDO-Me, seems to be an interesting and quite extensively investigated compound. That semisynthetic derivative, containing a cyano group at the C-2 position (Figure 4), significantly inhibited the growth of several ovarian cancer cell lines by increasing the rate of apoptosis [43]. Its effectiveness was confirmed in vivo with the use of the HO8910 and SK-OV-3 murine xenograft models [44]. BM-induced cell death was preceded by mitochondrial depolarization and cleavage of procaspases-3, -8, and -9.

Other oleanane-type PTs exhibiting anti-proliferative and proapoptotic effects against ovarian cancer cells include glycyrrhetinic acid [45], bartogenic acid [46], and gypsogenin [47]. Notably, the first of these compounds also demonstrated the ability to inhibit angiogenesis [48]. On the other hand, some plant-derived oleanane-type triterpenoid saponins, such as theasaponins or avicins, turned out to be quite efficient inhibitors of proliferation and inducers of cell death in ovarian cancer cell lines [33,49].

Ursane is an isomer of oleanane in which one of the methyl groups has been shifted from the C-20 position of ring E to the C-19 position (Figure 3B). A widely known and best-studied representative of ursane-type PTs is ursolic acid (UA) [24]. UA has been identified in a large number of botanical resources and has demonstrated relatively strong anti-cancer activity. It was the most potent inhibitor of cancer cell growth among several PTs extracted from *Betula utilis* bark. UA was also found to be highly selective for cancer cells over nontumorigenic cells [50]. Interestingly, ovarian cancer cell lines were found to be among the most sensitive to the cytotoxic effects of UA [50,51]. UA induced mitochondrial-dependent apoptosis in SK-OV-3 cells by altering the balance between pro-apoptotic and anti-apoptotic proteins [52]. It has been postulated that additional mechanisms of its action on ovarian cancer cells include reducing resistance to chemotherapeutic drugs [53], suppressing cell migration, and attenuation of cancer stem cells [54]. Other ursane-type PTs that exhibited anti-cancer activity against ovarian cancer cells include asiatic [55], pomolic [56], and corosolic acids [57]. Asiatic acid (AA) has become a quite popular subject of research. It is typically isolated from the medicinal plant *Centella asiatica* and exerts a complex impact on cancer cell biology. AA has been shown to inhibit epithelial-to-mesenchymal transition and colony formation as well as induce cell cycle arrest and apoptosis in some ovarian cancer cell lines [58]. Conversion of AA (as well as some other PTs) into rhodamine B conjugates resulted in mitocans that exerted strong cytotoxicity against ovarian cancer cells, with IC_50_ values in the nanomolar range [59].

Lupane-type compounds are the third important group of PTs, with lupeol, betulin, and betulinic acid as the most prominent representatives (Figure 3C). Their characteristic feature is the structure of the E ring, which consists of only five carbon atoms. Betulin is widely distributed in plants, but the outer bark of birch trees is a particularly abundant source of that PT (up to 30% of dry weight). Betulin turned out to be quite an efficient apoptosis inducer in both ovarian cancer cell lines and primary cultures of neoplastic cells [60,61]. Nevertheless, betulinic acid (BA) is believed to be a more efficient anti-neoplastic agent. BA exerted dose-dependent anti-proliferative and pro-apoptotic effects in several ovarian cancer cell lines (e.g., A2780, SK-OV-3, OVCAR-3, OVCAR-5, IGROV-1). Mechanistically, it predominantly activated the mitochondrial pathway, which was associated with loss of mitochondrial membrane potential, upregulation of Bcl-2-associated X (Bax), downregulation of Bcl-2, as well as activation of caspase-9 and -3, ultimately leading to cell demise [62,63,64]. BA suppressed migratory and invasive capacities of ovarian cancer cells both in vitro and in vivo, suggesting its possible anti-metastatic activity. That PT reduced tumor growth in murine ovarian cancer model, thus providing proof-of-principle for BA anti-tumor activity beyond cell culture [65]. Several authors have reported chemosensitizing action of BA and its synergistic effects with some cytostatics [66,67]. Some interesting anti-cancer action was observed also in the case of lupeol [68]. Chodurek et al. [60] found that chemical modification of betulin by introducing a propynoyl group at carbon C-28 (Figure 4) yielded a series of derivatives with markedly enhanced cytotoxicity against ovarian cancer cell lines. What is more, the authors observed induction of necrotic-like cell death preceded by dissipation of the mitochondrial potential in SK-OV-3 and OVCAR-3 cells after treatment with these acetylenic derivatives of betulin.

Among the other structural categories of PTs, significant attention is given to the friedelane-type compounds: celastrol and pristimerin (Figure 3D). They were quite efficient in growth inhibition and apoptosis induction in several ovarian cancer cell lines [69,70]. Taraxastanes such as taraxasterol are believed to play a key role in anti-cancer activity of extracts from *Taraxacum officinale* (Figure 3E) [71], whereas the multiflorane-type PT—karounidiol, isolated from the seeds of *Trichosanthes kirilowi*—is considered as a potential anti-tumor promoter (Figure 3F) [72]. Glutinol, a member of the glutynane-type PTs, has also been reported to exert an interesting influence on the biology of ovarian cancer cells. The IC_50_ of glutinol in OVACAR-3 neoplastic cell line was 6 µM [73].

**Figure 5:**
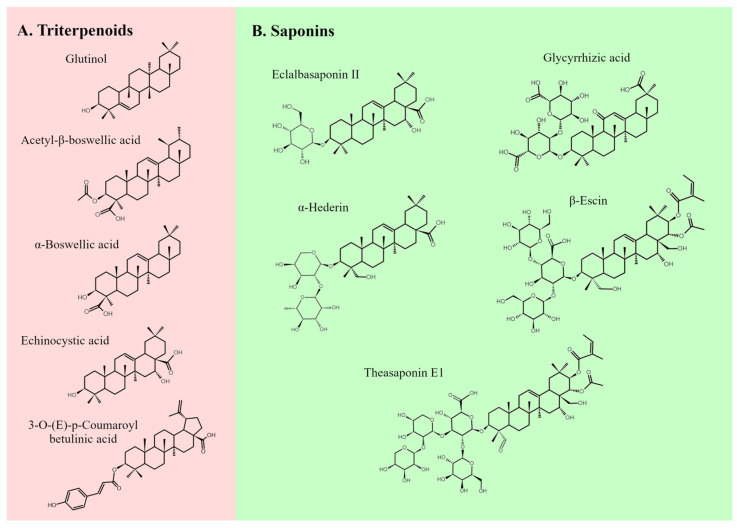
Selected triterpenoids and saponins with documented inhibitory activity against cancer cells.

Terpenoids occur in the plant raw material both as free form and as conjugated molecules. Glycosylated derivatives of PTs are known as triterpenoid saponins (Figure 5) [24].

## 4. Molecular Mechanisms of Pentacyclic Triterpenoids’ Action on Ovarian Cancer Cells

### 4.1. The PI3K/AKT/mTOR Signaling Pathway

PI3Ks constitute a family of heterodimeric lipid kinases composed of a catalytic p110 subunit and a regulatory p85 subunit that recruits the complex to activated receptor tyrosine kinases or G protein-coupled receptors (GPCRs). Once activated, PI3K converts phosphatidylinositol-4,5-bisphosphate (PIP2) into phosphatidylinositol-3,4,5-trisphosphate (PIP3), which brings phosphoinositide-dependent protein kinase-1 (PDK1) and AKT to the membrane. The tumor suppressor, PTEN, reverses this process by dephosphorylating PIP3. AKT becomes fully activated after phosphorylation by PDK1 (Thr308) and mechanistic target of rapamycin complex (mTORC2) (Ser473), enabling it to regulate mTORC1, protein synthesis, metabolism, cell growth, and survival pathways. In parallel, AKT modulates cell survival pathways and influences feedback loops impacting both mTORC1 and mTORC2 activities [74].

**Figure 6:**
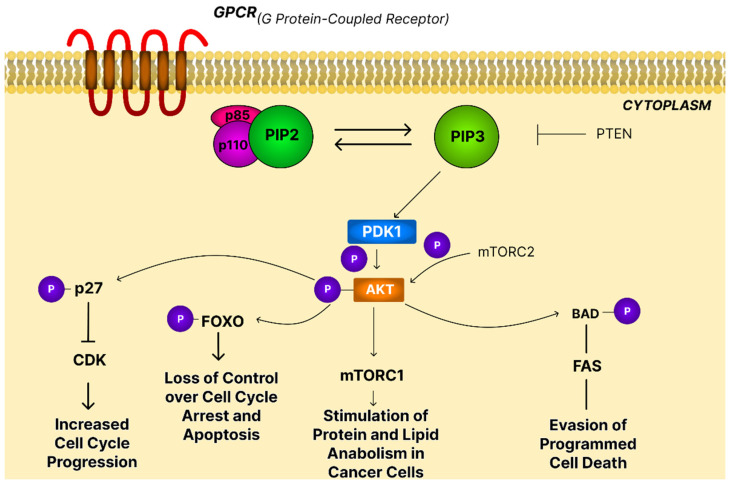
Simplified diagram of the PI3K/AKT pathway. Activation of PI3K kinase first leads to the phosphorylation of PIP2 and then AKT. Activated AKT phosphorylates a broad spectrum of substrates, including p27, FOXO, and BAD, involved in cell cycle progression and apoptosis. Furthermore, it also stimulates mTORC1 signaling, which stimulates protein and lipid synthesis in cells. PI3K activity can be inhibited by the dephosphorylating action of PTEN. The arrow and line indicate a stimulating effect. The lines ending with perpendicular bars indicate an inhibition.

Aberrant activation of the PI3K/AKT pathway promotes oncogenesis by supporting proliferation, blocking apoptosis, and reprogramming metabolism. AKT inactivates tumor suppressors (Forhead box O (FOXO), p27), inhibits pro-apoptotic proteins (Bcl-2-associated agonist of cell death (BAD), procaspase-9). Furthermore, it inhibits TNF-related apoptosis-inducing ligand/apoptosis ligand 2 (TRAIL/APO-2L)-induced apoptosis, contributing to therapeutic resistance. Oncogenic PI3K/AKT signaling drives metabolic reprogramming to sustain tumor growth by promoting glucose and nutrient uptake and activation of glycolytic and lipogenic enzymes through mTORC1 activation (Figure 6) [75].

In OC, this pathway is frequently altered. Activation of this pathway is observed in approximately 70% of OC cases, often driven by phosphatidylinositol-4,5-bisphosphate 3-kinase catalytic subunit alpha (PIK3CA) mutations or PTEN loss, leading to uncontrolled PI3K activity and excessive AKT phosphorylation. These alterations, especially common in endometrioid and clear cell ovarian cancers, lead to excessive AKT activation and correlate with poorer survival, chemotherapy resistance, and enhanced cancer stem cell traits that drive recurrence and aggressiveness [14].

Changes in PI3K/AKT/mTOR activity are primarily the results of modified phosphorylation status of key proteins. Yang et al. [61] demonstrated that incubation of OVCAR-3 cells with betulin led to a significant decrease in levels of phosphorylated p-PI3K, p-AKT, p-mTOR, resulting in their limited functionalities. Ren et al. [55] observed a similar drop in phosphorylation status in OVCAR-3 and SK-OV-3 cells after treatment with asiatic acid. The direct effect of AA on protein modification was demonstrated by overexpression of constitutively active enzyme prior to the incubation with the acid, which significantly reduced the observed cytotoxic effect.

Activation of the AKT/mTOR/ribosomal protein S6 kinase (S6K) pathway is thought to inhibit cellular autophagy. It was observed that OA administration reduced the expression of phosphorylated components of this pathway, leading to attenuation of the signaling cascade, ultimately resulting in autophagy induction in MCF10A cells. This phenomenon had no effect on the viability of normal MCF10A cells; however, it resulted in growth inhibition of the KRAS-transformed derivative of this cell line. Reactivation of the AKT/mTOR/S6K pathway by Insulin-like growth factor 1 (IGF-1) led to a reduction in the conversion of microtubule-associated protein 1A/1B-light chain (LC3)-I to LC3-II and Beclin-1 expression, which were involved in the formation and maturation of autophagosomes, in OA-treated cells. This allowed the following conclusion that suppressing the AKT/mTOR/S6K signaling pathway is necessary for autophagy induction [76]. Zhang et al. [77] showed that UA not only reduced the Beclin-1 and LC3 expression and inhibited the PI3K/AKT/mTOR pathway but also enhanced the Bax expression and decreased Bcl-2 expression that finally resulted in reduced autophagy and increased proapoptotic activity of SK-OV-3 cells.

Many pentacyclic triterpenes show proapoptotic activity. Yao et al. [78] assessed the effect of taraxerol on the PI3K/AKT/mTOR signaling pathway and apoptosis process in LPS-induced RAW264.7 cells and HeLa cells. The decline in PI3K and AKT protein phosphorylation status was dose-dependent. Suppressing the PI3K/AKT/mTOR signaling was associated with the induction of apoptosis through reducing the Bcl-2/Bax ratio. The effect of alpha-hederin, the active saponin of *Nigella sativa*, on apoptotic activity of cells were also investigated [79]. The incubation of SK-OV-3 with alpha-hederin resulted in the initiation of the mitochondrial pathway of apoptosis. The increased levels of caspase-9 and -3 after alpha-hederin administration were also observed in SCC-25 oral cancer cells. In the case of both experiments, observed results were closely related to the suppression of the PI3K/AKT/mTOR signaling pathway [80]. Cho et al. [81] evaluated the effect of echinocystic acid and its glycosides, eclalbasaponin I and II, isolated from *Eclipta prostrata* L., on SK-OV-3, A2780, and OVCAR-3 ovarian cancer cells and Hec1A and ISHIKAWA endometrial cancer cells. The eclalbasaponin II (Figure 4) was characterized by the highest anti-proliferative and proapoptotic activity. Additionally, the treated cells revealed features of autophagy concomitantly with inhibition of the mTOR signaling. To confirm the relationship between apoptosis, autophagy and mTOR signaling in saponin-treated ovarian cancer cells, the MHY1485 (a mTOR activator) was administrated. This compound reversed eclalbasaponin II-induced growth inhibition, LC3-II accumulation, and mTOR phosphorylation. miRNA-21, a molecule potentially suitable as a marker for some cancer types, can augment signaling in the PI3K/AKT/mTOR pathway. Celastrol has been shown to effectively induce apoptosis through the downregulation of miRNA-21 and the PI3K/AKT signaling pathway in both ovarian and gastric cancer cells [70,82]. That compound was able to suppress the described pathway and induce programmed cell death also in melanoma cells [83].

### 4.2. The MAPK/ERK Signaling Pathway

**Figure 7:**
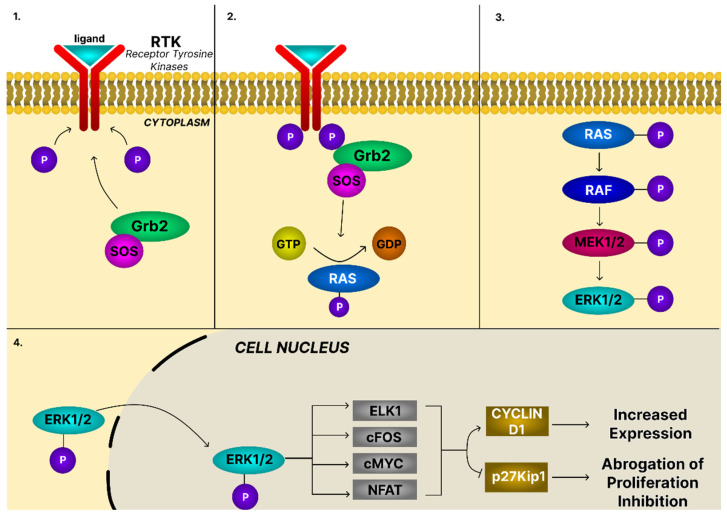
Simplified diagram of the MAPK pathway. Ligand binding RTKs (**1**) induces dimerization and autophosphorylation, recruiting adaptor proteins such as Grb2 and SOS to activate Ras (**2**). Active Ras triggers the Raf–MEK–ERK cascade (**3**), culminating in ERK activation and nuclear translocation, where ERK regulates transcription factors to drive gene expression for proliferation, differentiation, and metabolism (**4**). The arrow indicates a stimulating effect. The line ending with perpendicular bar indicates an inhibition.

The Ras/Rapidly accelerated fibrosarcoma (Raf)/Mitogen-activated protein kinase (MEK)/ERK cascade is a canonical MAPK pathway that integrates extracellular signals to regulate proliferation, differentiation, survival, and stress adaptation. Activation begins with ligand binding to receptor tyrosine kinases (RTKs) and triggering Ras activation. Active Ras subsequently engages Raf kinases, which phosphorylate MEK1/2, leading to ERK1/2 activation. Activated ERK translocates to the nucleus, where it modulates transcription factors, such as ELK1, c-Fos, c-Myc, and nuclear factor of activated T-cells (NFAT), thereby driving gene expression programs essential for proliferation, differentiation, and metabolic adaptation (Figure 7).

ERK activity is tightly controlled by scaffold proteins, negative feedback mechanisms, and dual-specificity phosphatases (DUSPs), and dysregulation causes constitutive ERK signaling associated with cancer [84]. Persistent ERK activation drives oncogenesis by promoting G1-S cell cycle progression, enhancing proliferation, and inducing epigenetic changes that silence tumor suppressor genes [84].

In OC, MAPK/ERK signaling contributes to tumor growth, invasion, metastasis, and chemoresistance. Its activation arises either from mutations in core signaling genes (KRAS, BRAF, NRAS) or from overexpression of growth factor receptors such as epidermal growth factor receptor (EGFR) and human epidermal growth factor receptor 2 (HER2), which trigger phosphorylation cascades culminating in ERK1/2 activation. ERK facilitates extracellular matrix degradation and peritoneal dissemination via matrix metalloproteinases (MMP-2, MMP-9) and cytoskeletal remodeling and promotes EMT through induction of transcription factors such as Snail, Slug, and ZEB1, thereby increasing motility and invasiveness. ERK also enhances resistance to chemoresistance by upregulating anti-apoptotic proteins (Bcl-2, Mcl-1) and activating survival pathways such as PI3K/AKT [15].

Given its central role in shaping of ovarian cancer cell phenotype, the MAPK/ERK pathway represents an attractive therapeutic target. A number of PTs have been reported to suppress the activity of protein kinases constituting this system in cancer cells. Wang et al. [84] demonstrated that the concentration-dependent inhibition of CAOV3 cell growth and induction of apoptosis by ursolic acid were accompanied by inhibition of ERK activity. The authors further reported that UA increased the expression of MKP-1, an enzyme responsible for ERK inactivation. Li et al. [48] reported that glycyrrhetinic acid not only decreased the level of phosphorylated ERK in the ovarian cancer A2780 cell line but also in human umbilical vein endothelial cells (HUVECs). In view of its capacity to diminish VEGF-induced HUVEC activity, the modulation of the MAPK/ERK pathway by GA may represent an important mechanism underlying the inhibition of angiogenesis in ovarian tumors. Investigations across a range of cancer cell lines from different tissue origins have demonstrated the ability of lupeol [85], betulinic acid [86], oleanolic acid [87], asiatic acid, celastrol [88], taraxerol [89], multiflorane [90] to inhibit the activity of the MAPK/ERK pathway. Interestingly, according to Kim et al. [91], the modulation of this pathway by oleanolic acid was cell line-dependent: the compound decreased p-ERK levels in DU145 cells but elevated them in MCF-7 and U87 cells. Despite these differences, OA induced cytostatic and proapoptotic effects in all three cell types. It has been demonstrated that activation of the MAPK/ERK pathway in some cancer cell lines was necessary for the induction of apoptosis by oleanolic [92] and ursolic [93] acids. In particular, ursolic acid has been reported to upregulate the phosphorylation of ERK in SK-OV-3 ovarian cancer cells. Similarly, AA increased p-ERK levels in some breast cancer cell lines [94]. The results outlined above are consistent with the concept of ERK kinases functioning as a double-edged sword in cancers. Most probably, the role of ERK kinase in regulation of cancer cell apoptosis largely depends on the cellular context, kind of stimulus, the strength and duration of pathway activation, and its interplay with other regulatory signals [95].

### 4.3. The NF-κB Signaling Pathway

**Figure 8:**
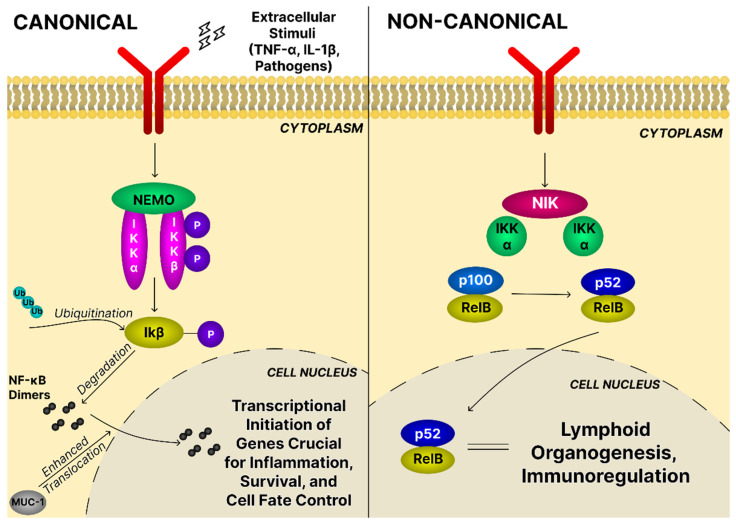
Simplified diagram of the canonical and noncanonical NF-κB pathways. Activation of the canonical pathway leads to phosphorylation of IKK and then IκB, followed by its ubiquitination and proteasomal degradation. This enables the release and translocation of NF-κB dimers to the nucleus and the regulation of gene expression associated with inflammation and cell survival. Activation of the noncanonical pathway, due to the activity of NIK and IKKα kinases, leads to translocation of the RelB/p52 complex to the nucleus, which influences lymphatic system development and immunoregulation. The arrow and line indicate a stimulating effect.

NF-κB regulates immune and inflammatory responses, cell survival, and oncogenic processes. It consists of transcription factors formed by combinations of RelA, RelB, c-Rel, p50, and p52, which are normally sequestered in the cytoplasm by inhibitors of the IκB family [96]. Diverse external signals—including pro-inflammatory cytokines, pathogen-associated molecular patterns (PAMPs), and growth factors—activate the IkappaB kinase (IKK) complex (IKKα, IKKβ, and NF-κB essential modulator (NEMO)). Activated IKK phosphorylates IκB, leading to its degradation and allowing NF-κB dimers to translocate into the nucleus and initiate transcription of target genes critical for inflammation, survival, and cell fate control [97]. Two mechanistically distinct NF-κB cascades are recognized: the canonical pathway, driven by IKKβ and NEMO that primarily activates p65/p50, and the noncanonical pathway that relies on NF-κB-inducing kinase (NIK) and IKKα to process p100 into p52, generating RelB/p52 complexes (Figure 8) [97].

In OC, NF-κB acts as a key transcriptional regulator that drives tumor initiation, progression, metastasis, and therapeutic resistance. Elevated NF-κB activity, frequently observed in high-grade serous ovarian carcinoma [98], integrates inflammatory cues, cytokine signaling, and pathways activated by oncogenic transformation into a unified regulatory network that sustains malignant proliferation and immune evasion [16]. Constitutive NF-κB signaling maintains continuous expression of anti-apoptotic genes, including Bcl-2, B-cell lymphoma-extra large (Bcl-xL), and members of the inhibitor of apoptosis (cIAP) family, providing ovarian cancer cells strong resistance to apoptosis and contributing to poor responses to platinum drugs and taxanes [12]. NF-κB promotes tumor invasion and metastasis by transcriptional activation of matrix metalloproteinases (e.g., MMP-9) and pro-metastatic chemokines such as CXCL8, which collectively remodel the extracellular matrix, promote epithelial–mesenchymal transition, and enhance peritoneal implantation capacity. NF-κB profoundly shapes the tumor microenvironment: it induces a pro-inflammatory secretome able to reprogram stromal and immune compartments toward a tumor-supportive phenotype, concomitantly with establishing an immuno-evasive microenvironment, e.g., by releasing programmed death-ligand 1 (PD-L1), a factor attenuating anti-tumor T-cell responses [98]. It is important to note that, because NF-κB controls many essential cellular processes, developing safe and selective therapies targeting this pathway remains a major challenge [99].

Dubey et al. [46] evaluated the effect of bartogenic acid in an SK-OV-3 xenograft mouse model. Bartogenic acid is a naturally occurring pentacyclic triterpenoid with numerous biological activities. Structurally, the tested compound is similar to ursolic acid and oleanolic acid, indicating the possibility of having similar biological properties, including primarily anti-cancer activity. Bartogenic acid either alone or combined with paclitaxel showed significant inhibition of cytoplasmic positivity of NF-κB in tumor tissues concomitantly with reduction in the number of viable tumor cells and the degree of necrosis. Pristimerin, another naturally occurring triterpenoid, has been shown to inhibit the proliferative activity of OVCAR-5, MDAH-2774, OVCAR-3, and SK-OV-3 ovarian cancer cells and induce the intrinsic apoptosis pathway by suppression of prosurvival signaling proteins, like p-AKT, p-mTOR, and NF-κB. The studied PT significantly decreased expression of NF-κB-regulated anti-apoptotic proteins as Bcl-2, Bcl-xL, c-IAP1, and survivin, thus reducing the cell resistance to apoptosis [69]. Inhibition of the expression of NF-κB and NF-κB-dependent anti-apoptotic proteins has also been observed as a result of the action of a synthetic derivative of oleanolic acid—CDDO-Me (Figure 4) by Gao et al. [100]. The compound caused oxidative stress in OVCAR-5 and MDAH-2774 ovarian cancer cells through the increased production of ROS, as H_2_O_2_, that mediated its anti-cancer, proapoptotic effects. The effect was abolished when a strong antioxidant, N-acetylcysteine, was added to the experimental design.

The NF-κB pathway may be a target for ursolic acid derivatives. Jiang et al. [101] synthesized some derivatives of UA containing additive moieties at the C-28 carbon. The results showed that the strength of the inhibitory effect on NF-κB activity in several different cancer lines (i.e., SK-OV-3, A549, HepG2, and T24) depended on the chain length. The greater inhibitory activity had a UA derivative with a diamide gallic acid moiety (Figure 4). The NF-κB inhibition occurred as a result of the interaction of the C-3 carbonyl group of the derivative with the active site of NF-κB involving hydrogen bonds. Treatment of cancer cells with that compound resulted in cell cycle arrest at the G1 phase, apoptosis induction, and suppressed migration. In the context of cancer cell migration and invasiveness, special attention should be paid to proteolytic enzymes, such as MMP-2 and -9, and signaling molecules involved in the EMT. Wang et al. [102] demonstrated the effect of celastrol on limiting the migration and invasion of OVCAR-3 and SK-OV-3 cancer cells. This was due to inhibition of the NF-κB signaling pathway by blocking IκBα phosphorylation and preventing IκBα degradation and p65 accumulation. Furthermore, celastrol inhibited the expression and activity of MMP-9, which suggests its effect on limiting invasiveness and metastasis. Another triterpene compound that has been shown to have an inhibitory effect on cell migration and invasiveness was β-escin. Kwak et al. [103] investigated the effect of β-escin, a triterpene saponin isolated from horse chestnut (*Aesculus hippocastanum*) seeds on melanoma. The results showed that β-escin inhibited the migration and motility of B16F10 and SK-MEL5 melanoma cells in a dose-dependent manner. At the expression level, an increase in the expression of tissue inhibitors of metalloproteinase (TIMP)-1 and -2 and an inhibition of NF-κB and IκB expression were observed.

Inhibition of IκK kinase and NF-κB expression, and thus the NF-κB signaling pathway, could also be observed in cancer cell cultures treated with boswellic acid. Trivedi et al. [104] evaluated the anti-tumor activity of boswellic acids isolated from *Boswellia serrata* in chemoresistant PC-3 prostate cancer cells. The administration of acetyl-β-boswellic acid and acetyl-11-keto-β-boswellic acid to in vitro PC-3 cultures resulted in the inhibition of cell proliferation and the promotion of apoptotic death. Studies conducted in mice indicated visible tumor reduction with no detectable systemic toxicity. In the study conducted by Shankar et al. [105], LNCaP and DU145 prostate cancer cell lines were used. Cells were treated with betulinic acid, which led to phosphorylation of IKKα and IκBα kinase, inhibition of nuclear localization and cytosolic accumulation of NF-κB/p65, and inhibition of DNA binding. The obtained results showed that in LNCaP cells, administration of BA increased Ser15 phosphorylation of p53 protein and its stabilization, leading to the induction of Bax and p21/WAF1 expression and thus apoptosis. In DU145 cells, the increase in p21/WAF1 levels occurred independently of p53. A decrease in Bcl-2 expression, increased cytochrome c release, and progression of apoptosis were also observed. Similarly, in the experiment carried out by Cheng et al. [106], inhibition of nuclear localization and cytosolic accumulation of NF-κB/p65 occurred as a result of the action of 2α,3β-dihydroxy-urs-12-en-28-oic acid (CRA) isolated from the root of *Actinidia valvata* Dunn on human gastric cancer cell line BGC823. The obtained results indicated a decrease in the expression levels of p65, Bcl-2, Fas, and second mitochondrial-derived activator of caspase (Smac) and an increase in the expression levels of IκBα, Bax, and survivin. Thus, the results obtained so far indicated that CRA, like many other triterpene compounds, had the ability to inhibit the NF-κB signaling pathway and exhibits significant anti-cancer potential.

### 4.4. The JAK/STAT Signaling Pathway

**Figure 9:**
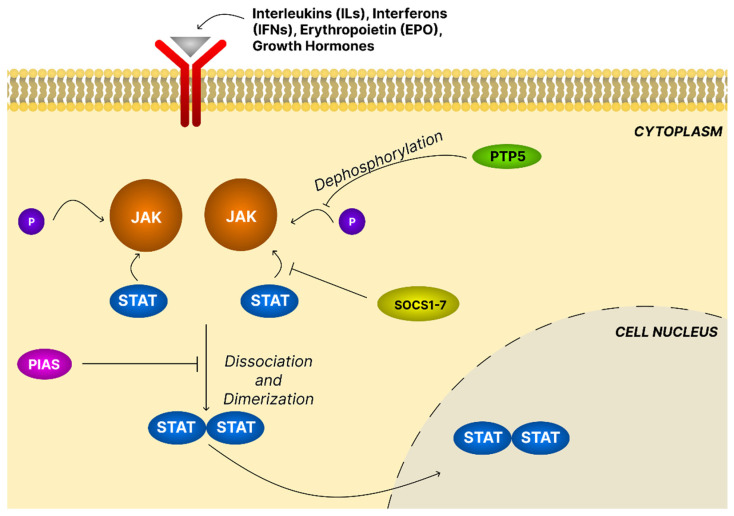
Simplified diagram of the JAK–STAT pathway. Ligand binding to cytokine or growth factor receptors (e.g., ILs, IFNs, Epo, growth hormones) induces receptor dimerization and activates receptor-associated JAKs. Activated JAKs phosphorylate receptor tyrosine residues, creating docking sites for STAT proteins. Phosphorylated STATs dimerize via Src homology 2 (SH2) domains and translocate to the nucleus, where they bind DNA and regulate transcriptional programs controlling immunity, proliferation, and differentiation. The arrow indicates a stimulating effect. The lines ending with perpendicular bars indicate an inhibition.

The JAK/STAT constitutes a highly conserved signaling cascade that directly couples extracellular cytokine and growth factor cues to nuclear transcriptional responses, thereby regulating immunity, proliferation, differentiation, and programmed cell death. Canonical activation begins with ligand binding of interleukins (IL-2, IL-3, IL-6, IL-10), interferons (IFN-γ), erythropoietin (Epo), or growth hormones, to cognate receptors, inducing receptor dimerization. Preassembled dimers facilitate rapid signal transduction. Receptor-associated JAKs undergo trans-phosphorylation and subsequently phosphorylate receptor tyrosine residues, generating docking sites for STATs. STATs then dissociate, dimerize, and translocate into the nucleus, where they bind DNA directly, interact with nonSTAT cofactors, and modulate chromatin accessibility to regulate gene expression programs (Figure 9).

Negative regulation ensures signaling fidelity. Suppressors of cytokine signaling (SOCS1–7) inhibit receptor–STAT interactions, directly inactivate JAKs, or promote ubiquitin-mediated degradation, while protein inhibitors of activated STATs (PIAS) restrict STAT dimerization and DNA binding. Protein tyrosine phosphatases (PTPs) further attenuate activity by dephosphorylating JAKs and STATs. Perturbations of these checkpoints, whether through receptor overexpression or oncogenic mutations, foster persistent pathway activity and underlie chronic inflammation, autoimmunity, and malignant transformation [107].

In OC, aberrant JAK/STAT signaling is a defining feature of disease progression, underpinning proliferation, survival, angiogenesis, metastasis, and immune escape. Hyperactivation of STAT3, in particular, correlates with aggressive clinicopathological features and unfavorable prognosis. The interaction between Fc gamma binging protein (FCGBP) and the NF-κB p65 subunit elevates IL-6 secretion, activates JAK1/2, and phosphorylates STAT3. Activated STAT3 dimers translocate to the nucleus, driving transcription of several genes, including VEGF, Bcl-2, and Myc [17]. STAT3-driven transcriptional programs promote immunosuppression and facilitate immune evasion. STAT3 additionally regulates metabolic reprogramming supporting tumor growth and metabolic adaptation [108].

Pentacyclic triterpenoids such as ursolic acid, celastrol, and betulinic acid have been shown to modulate the JAK/STAT3 signaling pathway, which may underlie their anti-inflammatory, anti-cancer, and cardioprotective effects. Ursolic acid effectively suppresses JAK2 and STAT3 phosphorylation in various cellular models. Zhou et al. [107] demonstrated that in NRK-52E rat kidney cells exposed to high glucose, ursolic acid reduced the elevated levels of p-JAK2 and p-STAT3, while activation of the JAK2–STAT3 pathway markedly diminished the anti-ferroptotic and antioxidant effects of ursolic acid. Similarly, Fan et al. [109] reported that treatment of A549 lung adenocarcinoma cells with UA or its nanoparticles significantly decreased STAT3 expression. The study demonstrated that the inhibition of cell viability by UA was associated with suppression of the STAT-3 signaling pathway. In nonsmall-cell lung cancer A549 and H460 cells, ursolic acid suppressed the phosphorylation of EGFR, JAK2, and STAT3 and downstream PD-L1 expression. The authors suggested that the UA may exert its effects through the EGFR/JAK2/STAT3/PD-L1 signaling pathway. These findings highlighted STAT3 as a key mediator of the anti-cancer activity of ursolic acid, with STAT3 serving as a crucial link between EGFR signaling and PD-L1 expression [110]. In colorectal cancer HCT116 and HT29 cells, ursolic acid similarly decreased STAT3 phosphorylation and nuclear translocation. The presented results indicated that UA promoted apoptosis in colorectal cancer cells through upregulation of miR-4500 and suppression of STAT3 phosphorylation [111]. Ye et al. [112] identified STAT3 in rat cardiomyocytes as a direct molecular target of celastrol, with the compound binding to both the SH2 domain and the coiled-coil domain of STAT3, leading to decreased STAT3 tyrosine phosphorylation and impaired nuclear translocation, respectively. Betulinic acid also exerted potent STAT3 inhibitory effects. Pandey et al. [113] showed that in multiple myeloma U266 cells, BA suppressed constitutive STAT3 activation, nuclear translocation, and DNA-binding activity. Betulinic acid inhibited constitutive phosphorylation of JAK1 and constitutive activation of JAK2 in U266 cells. The inhibition of STAT3 activation by BA was mediated through tyrosine phosphatase activity. Moreover, forced STAT3 overexpression markedly reduced betulinic acid-induced apoptosis in A293 cells. Collectively, these findings highlight pentacyclic triterpenoids as effective modulators of the JAK/STAT3 signaling pathway with promising therapeutic potential especially in cancer.

### 4.5. The Notch Signaling Pathway

**Figure 10:**
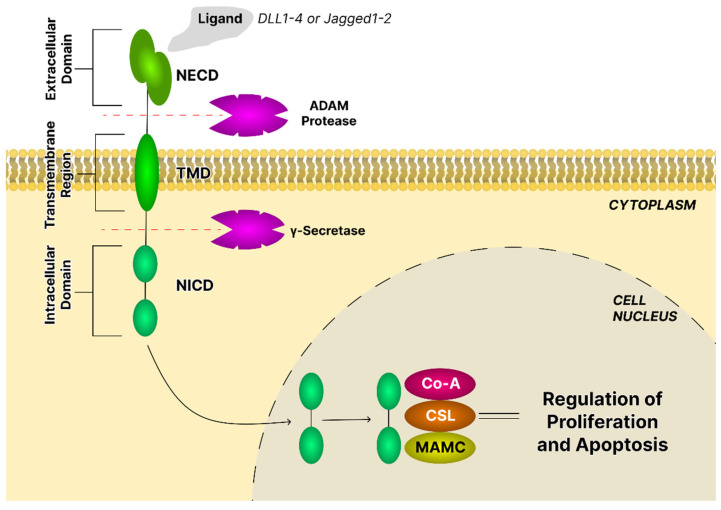
Simplified diagram of the canonical Notch pathway. Binding of a Delta-like or Jagged family ligand to the Notch receptor leads to its shortening by ADAM protease cleavage. Subsequently, γ-secretase releases NICD, which translocates to the cell nucleus, forming a transcriptional complex with CSL and MAML that affects the expression of genes regulating cell proliferation and apoptosis. The arrow and line indicate a stimulating effect.

The Notch pathway is an evolutionarily conserved intercellular communication system that directly couples ligand engagement at the cell surface to transcriptional reprogramming within the nucleus. Notch receptors are single-pass transmembrane heterodimers consisting of an extracellular domain (NECD), a transmembrane region (TMD), and an intracellular domain (NICD). Receptor maturation involves glycosylation in the endoplasmic reticulum and S1 cleavage in the Golgi apparatus, yielding a heterodimer that is trafficked to the plasma membrane. Canonical signaling is initiated when ligands of the Delta-like (DLL1, DLL3, DLL4) or Jagged (Jagged1, Jagged2) families bind the receptor, triggering ADAM-mediated S2 cleavage and γ-secretase-mediated S3 cleavage. The released NICD translocates into the nucleus to form a transcriptional complex with Supressor of Hairless (CSL) and Mastermind-like (MAML) proteins and induces target genes such as Hes and Hey family members, which regulate cell proliferation, lineage specification, and apoptosis (Figure 10) [31].

Beyond canonical activation, Notch signaling also functions through noncanonical mechanisms that bypass ligand binding, CSL, or γ-secretase activity. These alternative modes enable extensive crosstalk with oncogenic and inflammatory networks, including Wnt/β-catenin, JAK/STAT, PI3K/AKT/mTOR, and NF-κB [114].

The role of Notch signaling in cancer is context-dependent and bidirectional. Activating mutations of Notch1 drive oncogenesis in T-cell acute lymphoblastic leukemia through upregulation of Myc and NF-κB, while in breast cancer, nonsmall-cell lung cancer, and gliomas, Notch receptors promote proliferation, apoptosis resistance, and cancer stem cell maintenance. Conversely, in cutaneous squamous cell carcinoma, Notch1 functions as a tumor suppressor, preserving epidermal stem cell homeostasis and promoting terminal differentiation [115].

Overexpression of Notch pathway components, including Notch1, Notch2, Notch3, Jagged ligands, and downstream effectors such as Hes1, has been consistently observed in high-grade ovarian carcinomas and correlates with poor prognosis. Mechanistically, hyperactive Notch1 induces EMT, enhancing migration and invasion, while Notch1–DLL4 interactions promote angiogenesis. Importantly, Notch3 has emerged as a critical regulator of CSCs, maintaining platinum resistance and tumor-initiating capacity. Specifically, NICD3 overexpression induces CD44 expression, reinforcing stemness phenotypes and facilitating tumor recurrence following chemotherapy. Additionally, Notch-driven metabolic reprogramming, especially increased glycolysis and adaptation to hypoxia, supports ovarian tumor survival and recurrence [18].

Ahmad et al. [116] assessed the potential of glycyrrhizic acid to block the Notch cascade and thus exert an anti-proliferative effect on HPV16+ CaSki cervical cancer cells. Incubation of cells with glycyrrhizic acid inhibited the Notch cascade by inhibiting the Notch1–Jagged-1 interaction, which under standard conditions leads to proteolysis and activation of target gene expression. As a result of inhibiting the Notch cascade, glycyrrhizic acid exerted an anti-proliferative effect. This effect was mediated by increased p21/WAF1 protein expression and, consequently, decreased CDK4 and cyclin D1 expression, ultimately leading to cell cycle inhibition and arrest of cancer cells at the G0/G1 transition. Xiong et al. [117] using murine RAW264.7 macrophages observed that asiatic acid, due to its ability to regulate the expression of not only Notch3 but also DLL4, may be a small molecule inhibitor of the Notch signaling pathway.

So et al. [118] assessed the effect of an oleanoic acid derivative, 2-cyano-3,12-dioxooleana-1,9(11)-dien-28-oic acid imidazolide (CDDO-Im), on triple-negative breast cancer (TNBC), SUM159 cell line. CDDO-Im (Figure 4) is one of the most potent synthetic triterpenoids, inducing growth inhibition and apoptosis in various human cancer cells, including, e.g., multiple myeloma, lung cancer, and pancreatic cancer. The effect of imidazole on the levels of proteins involved in the Notch and transforming growth factor (TGF-β)/Smad signaling pathways was assessed. Previous studies have highlighted the role of as many as four Notch receptors (1–4) in the development of breast cancer. Silencing Notch1 or Notch4 inhibited the ability of breast cancer cells to self-renew and form tumor spheres. Increased Notch3 activity was associated with aggressive human inflammatory breast cancer and increased lymphatic invasion. Interestingly, the role of the Notch2 receptor appeared to be opposing, with high levels suppressing cell growth and inducing apoptosis in TNBC cells. This experiment demonstrated that CDDO-Im increased Notch2 protein levels while simultaneously inhibiting the expression of Notch1 and Notch3, suggesting differential regulation of Notch receptors, which may be useful in developing new therapeutic strategies aimed at inhibiting this signaling pathway. In MBA-MD-231 and T47D breast cancer cells, Kushwaha et al. [119] investigated the possibility of inhibiting the Notch pathway after administration of 3-O-(E)-p-coumaroylbetulinic acid. In this case, a significant reduction in Notch signaling was evidenced due to decreased expression of Notch target effector genes, i.e., Hes1 and Hey1, in both lines. These genes encode proteins that act as transcription regulators, especially repressors.

### 4.6. The HIF-1α Signaling Pathway

**Figure 11:**
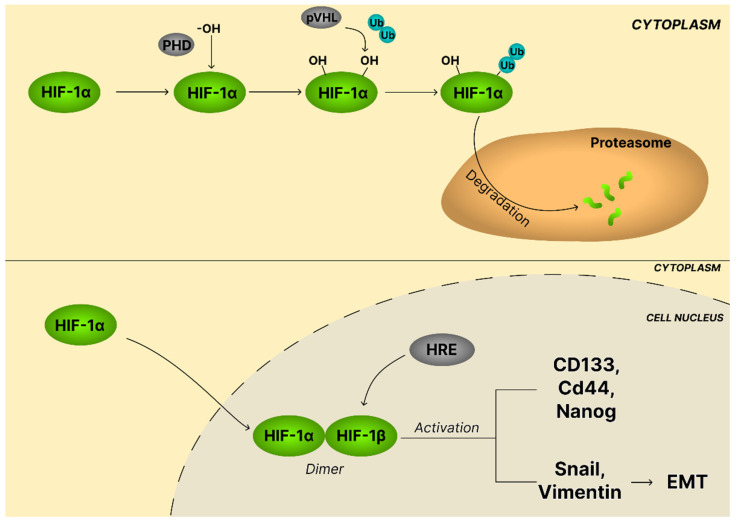
Simplified diagram of the HIF-1α pathway. Under normoxic conditions, HIF-α subunits are hydroxylated by PHDs, enabling recognition by the pVHL ubiquitin ligase complex and proteasomal degradation (**upper** part). During hypoxia, hydroxylation is suppressed, stabilizing HIF-α, which translocates to the nucleus, dimerizes with HIF-1β, and binds HREs to activate genes essential for cellular adaptation to low oxygen (**lower** part). The arrow and line indicate a stimulating effect.

Hypoxia-inducible factors (HIFs) are pivotal transcription factors composed of one of three oxygen-sensitive α-subunits (HIF-1α, HIF-2α, or HIF-3α) and a constitutively expressed β-subunit (HIF-1β/aryl hydrocarbon receptor nuclear translocator (ARNT)). Among these, HIF-1α is broadly expressed in most tissues under hypoxic conditions, whereas HIF-2α and HIF-3α display tissue-specific expression patterns [120]. Under normoxia, HIF-α subunits undergo prolyl hydroxylation by oxygen- and iron-dependent prolyl hydroxylases (PHDs), which promotes their recognition by the von Hippel–Lindau (pVHL) ubiquitin ligase complex and subsequent proteasomal degradation. Hypoxia suppresses this hydroxylation, thereby stabilizing HIF-α, which translocates to the nucleus, heterodimerizes with HIF-1β, and binds hypoxia-response elements (HREs) within target gene promoters to drive the transcription of hundreds of genes required for hypoxic adaptation (Figure 11). Importantly, HIF stabilization may also occur through hypoxia-independent mechanisms, including oncogene activation, tumor suppressor loss, NF-κB-driven transcriptional induction, and accumulation of oncometabolites such as lactate, fumarate, succinate, and 2-hydroxyglutarate. These alterations facilitate tumor progression by sustaining HIF stability and transcriptional activity [120].

In OC, hypoxia-driven HIF-1α signaling enhances tumor aggressiveness by promoting CSC-like traits, chemoresistance, and EMT. Under low oxygen, SK-OV-3 and HO8910 cells upregulate CSC markers (CD133, CD44, Nanog) and EMT markers (Vimentin, Snail) while suppressing E-cadherin, leading to increased tumorigenicity and drug resistance. Knockdown of HIF-1α or its downstream effector sirtuin 1 (SIRT1) reverses these effects, restoring chemosensitivity and epithelial characteristics [121].

Several studies have demonstrated that pentacyclic triterpenoids exert significant inhibitory effects on the HIF-1α signaling pathway, a critical regulator of tumor adaptation to hypoxic stress and angiogenesis. Ursolic acid was reported to significantly decrease HIF-1α and adenosine triphosphate (ATP)-binding cassette efflux transporter G2 (ABCG2) expression in SK-OV-3 ovarian cancer cells under hypoxic conditions. The study further suggested that suppression of the PI3K/AKT pathway contributes to the ursolic acid-mediated downregulation of HIF-1α [122]. Similarly Li et al. [123] found that theasaponin E1 suppressed HIF-1α protein expression in OVCAR-3 cells. In this study, the authors showed the ataxia telangiectasia mutated (ATM)/AKT/HIF-1α signaling axis plays a key role in the anti-cancer effects of theasaponin E1. Additionally, the presented data evidenced theasaponin E1 modulation of HIF-1α through both the PI3K/AKT and Notch1 pathways.

### 4.7. The TGF-β Signaling Pathway

**Figure 12:**
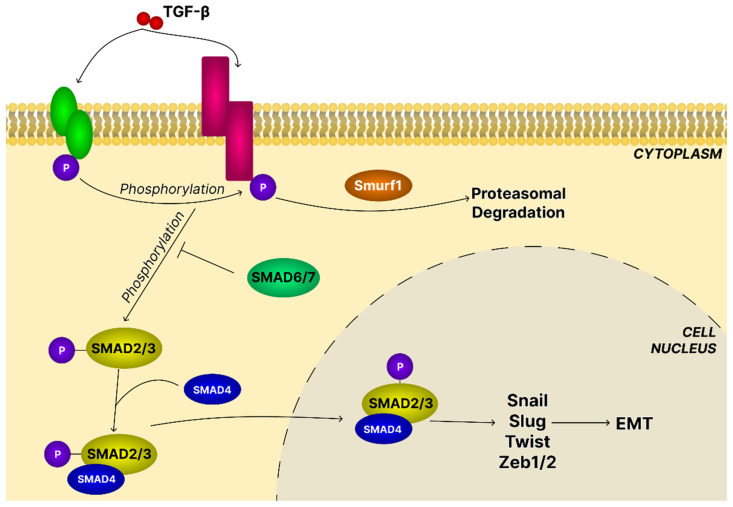
Simplified diagram of the canonical TGF-β pathway. Ligand binding to the receptor phosphorylates the Smad2/3 complex, which, upon association with Smad4, translocates to the nucleus and regulates transcription of target genes involved in EMT. The Smad 6/7 complex can inhibit Smad 2/3 phosphorylation. In turn, Smurf-1 activity causes proteosomal degradation of the receptor. The arrow indicates a stimulating effect. The line ending with perpendicular bar indicates an inhibition.

Activation of latent TGF-β requires dissociation of mature TGF-β from its latency-associated peptide (LAP), enabling receptor binding. This process occurs through acidification or modification by ROS in vitro and via proteolytic cleavage by extracellular matrix (ECM) proteases such as plasmin, MMPs, Cathepsin D, and Thrombospondin-1 (TSP-1) in vivo. TGF-β also forms large latent complexes (LLCs) with Latent transforming growth factor beta binding protein (LTBP) and Glycoprotein A repetitions predominant (GARP), which regulate its availability and activation. Integrins αvβ6 and αvβ8 activate TGF-β through mechanical deformation of the LLCs through Arg-Gly-Asp (RGD) motifs in LAP [124]. TGF-β is a pleiotropic cytokine within the TGF-β superfamily, which also includes activins, bone morphogenetic proteins, and anti-Müllerian hormone, with TGF-β1 being the most abundant. It signals through canonical Smad-dependent pathways, where ligand binding activates the TGF-β type I and type II receptors (TβRI and TβRII), leading to Smad2/3–Smad4 complex formation and nuclear gene regulation (Figure 12), and through noncanonical Smad-independent pathways such as MAPK, PI3K/AKT, RHO/Rho-associated protein kinase (ROCK), and NF-κB cascades, regulating numerous cellular functions [125]. Through these diverse mechanisms, TGF-β integrates oncogenic and inflammatory signals within the tumor microenvironment [126].

In OC, TGF-β has context-dependent dual roles: it suppresses tumors in early stages but acts as an oncogenic driver in advanced disease. As cancer cells become resistant to its growth-inhibitory effects, TGF-β signaling shifts toward oncogenic pathways. TGF-β induces EMT through Snail, Slug, Twist, and ZEB1/2, downregulating E-cadherin, promoting mesenchymal phenotypes, and enhancing migration/invasion. EMT also enriches stem-like cell populations, driving heterogeneity and recurrence [125]. TGF-β also remodels the tumor microenvironment by activating cancer-associated fibroblasts, promoting ECM deposition, secreting pro-tumorigenic cytokines, and enhancing invasion and angiogenesis. It skews immunity toward an immunosuppressive state and amplifies resistance to anti-tumor immunity [125].

In the study by Gao et al. [127], all TGF-β isoforms (-1, -2, and -3) demonstrated the ability to inhibit growth while simultaneously enhancing EMT in OVCAR3 cells in a dose-dependent manner. Expression of the mesenchymal markers N-cadherin and claudin-1 increased, while occludin expression decreased accordingly. It is important to note that the migrating cells retained their epithelial shape and E-cadherin expression. Regardless of the isoform (TGF-β 1–3), expression of the E-cadherin repressor Snail remained low, while expression of ZEB1 increased.

Wang et al. [58] evaluated the effect of asiatic acid on the EMT phenomenon occurring in SK-OV-3 ovarian cancer cells. A key element of this process was the loss of E-cadherin and the increase of N-cadherin. Administration of AA limited the EMT process, as evidenced by a decrease in N-cadherin expression while simultaneously increasing E-cadherin expression at both the mRNA and protein levels. The study also observed that the expression of epithelial markers (like E-cadherin) was increased, while the expression of mesenchymal markers (like vimetin, N-cadherin, and ZEB1/2) was suppressed after triterpene administration. Also, in the case of A549 lung cancer cells, an inhibitory effect of asiatic acid on epithelial–mesenchymal transformation was observed [128]. Induction of EMT by TGF-β1 in A549 cells caused the cells to be spindle-shaped and exhibit stromal cell characteristics, while AA reversed that processes. By increasing E-cadherin expression and inhibiting the expression of Snail repressor, N-cadherin, and vimentin, it maintained the characteristics of epithelial cells and thus limited the phenomenon of EMT. Not only asiatic acid but also uvaol obtained from olive trees (*Olea europaea* L.) can inhibit TGF-β1-induced EMT. Patrícia Gonçalves Tenório et al. [129] observed that uvaol, which did not exhibit significant cytotoxic effects on A549 cells, inhibited changes in cell morphology indicative of EMT progression and migration. In addition to morphological observations, EMT inhibition was confirmed by assessing the decreased expression of mesenchymal markers such as vimentin, N-cadherin, and fibronectin. It was also hypothesized that uvaol prevents β-catenin translocation to the cell nucleus and reduces the level of ZEB1 protein induced by TGF-β1 in A549 cells. Similar changes in the expression of TGF-β1 signaling pathway components and EMT transition were observed by Zhang et al. [130] in HCT116 and HCT-8 colon cancer cells exposed to ursolic acid. Administration of UA to in vitro cell cultures significantly reduced their viability and reduced their ability to migrate and invade, resulting in the desired anti-cancer effect. This effect was the result of suppression of the expression of proteins associated with the TGF-β1 signaling pathway, i.e., total TGF-β1 protein levels, p-Smad/2/3, ZEB1, and N-cadherin. Not only ursolic acid but also oleanolic acid derivatives can influence TGF-β signaling. So et al. [118] assessed the effect of CDDO-Im on the TGF-β/Smad signaling pathway in SUM159 cells. CDDO-Im inhibited TGF-β-stimulated cell migration by altering the transport and turnover of TGF-β receptors. The obtained results also demonstrated significantly reduced activin mRNA levels and TGF-β receptors mRNA levels, as well as reduced pSmad2/3 protein levels. Investigating the effect of TGF-β signaling inhibition by CDDO-Im on EMT and growth of triple-negative breast cancer will be an interesting area of future research.

### 4.8. The Wnt/β-Catenin Signaling Pathway

**Figure 13:**
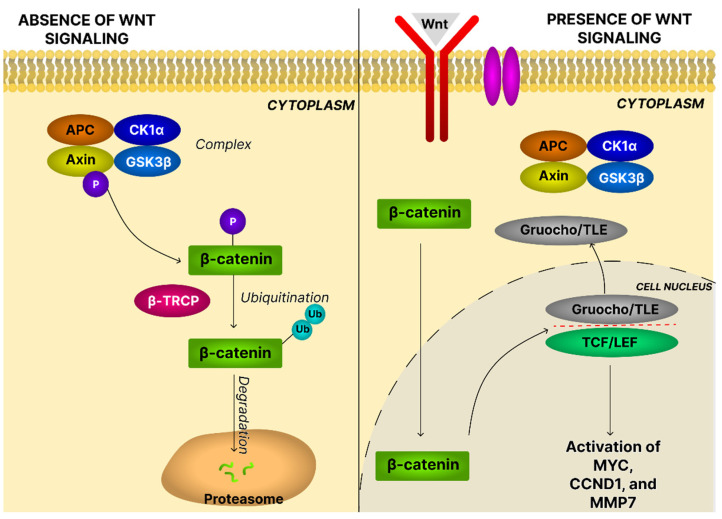
Simplified diagram of the Wnt/β-catenin pathway. In the absence of Wnt ligands, β-catenin is targeted for phosphorylation and proteasomal degradation by the destruction complex composed of APC, Axin, CK1α, and GSK3β. Upon Wnt binding to Fzd receptors and LRP5/6 co-receptors, the destruction complex is inhibited, allowing β-catenin stabilization and nuclear translocation. Nuclear β-catenin associates with TCF/LEF transcription factors replacing Groucho/TLE to activate target gene expression. The arrow indicates a stimulating effect.

The Wnt family constitutes a complex network of signal transduction pathways, broadly classified into canonical and noncanonical cascades. The canonical Wnt pathway, or Wnt/β-catenin signaling, relies on Wnt ligands, Frizzled (Fzd) receptors, and T-cell factor/lymphoid enhancer factor (TCF/LEF) transcription factors. After Porcupine (PORCN)-mediated acylation, Wnt ligands bind Fzd and Low-density lipoprotein receptor-related proteins 5 and 6 (LRP5/6) co-receptors, forming a ternary complex that recruits proteins such as Dishevelled (Dvl), Axin, and Glycogen synthase kinase-3 beta (GSK3β), to initiate signal transduction. β-Catenin is the principal nuclear effector. In the absence of Wnt stimulation, β-catenin is retained in a multiprotein destruction complex comprising adenomatous polyposis coli (APC), casein kinase 1α (CK1α), GSK3β, and Axin. This complex phosphorylates β-catenin, creating a recognition motif for the E3-ubiquitin ligase β-transducin repeat-containing protein (β-TRCP), leading to ubiquitination and proteasomal degradation. When Wnt signaling is active, this destruction complex is inhibited, allowing β-catenin to accumulate, enter the nucleus, and replace Groucho/Transducin-like enhancer of split (TLE) repressors on TCF/LEF to activate gene transcription (Figure 13) [131].

Noncanonical Wnt pathways, including Wnt/Planar Cell Polarity (PCP) and Wnt/Ca^2+^ signaling, operate independently of β-catenin. The Wnt/Ca^2+^ pathway modulates adhesion-related gene expression via intracellular calcium release, whereas the PCP pathway controls epithelial planar polarity and directional migration during development. These pathways are activated by noncanonical ligands such as Wnt5a or Wnt11 [131].

Inactivating mutations in components of the β-catenin destruction complex (APC, Axin, RNF43) lead to constitutive Wnt/β-catenin activation, disrupting cell adhesion, DNA repair, and apoptosis, thereby promoting tumorigenesis.

In OC, Wnt/β-catenin signaling plays a central oncogenic role by coordinating proliferation, invasion, cancer stem cell maintenance, and therapy resistance. Activation of the AKT → GSK3β → β-catenin axis promotes β-catenin accumulation and its nuclear activity. Wnt/β-catenin cooperates with other pathways, including AKT, to amplify proliferative and anti-apoptotic responses and facilitate adaptation to environmental stress. Cooperation between Wnt/β-catenin and pathways like AKT enhances tumor growth and survival, suggesting that effective therapy may require simultaneous targeting of multiple interconnected pathways [19].

Ursolic acid has been reported to inhibit Wnt/β-catenin signaling in different cancer models, including osteosarcoma and colorectal cancer. Zhang et al. [132] reported that in osteosarcoma 143B cells, UA suppresses β-catenin expression and reduces its protein levels in both the nucleus and cytoplasm, along with downregulation of its downstream targets c-Myc and cyclin D1. These results suggested that the anti-proliferative effect of ursolic acid on osteosarcoma cells may be linked to the inhibition of Wnt/β-catenin signaling. β-catenin knockdown potentiated, whereas its overexpression attenuated, the anti-proliferative and G1 arrest-inducing effects of ursolic acid. Notably, UA was also shown to upregulate p53 mRNA and protein expression levels and downregulate its negative regulator murine double minute 2 (MDM2), suggesting that Wnt pathway inhibition by ursolic acid may, at least in part, be mediated by p53 activation. Further supporting its role as a Wnt inhibitor, UA was also found to attenuate Wnt signaling in colorectal cancer SW620 cells. The compound significantly reduced mRNA and protein levels of key Wnt/β-catenin signaling molecules, including Wnt4, β-catenin, TCF4, and LEF1. Conversely, it increased the levels of total GSK-3β and phosphorylated β-catenin. This was accompanied by impaired nuclear translocation of β-catenin. The authors concluded that UA suppressed the Wnt/β-catenin signaling pathway, which contributed to its inhibitory effects on colorectal cancer cells in vitro [133]. Raddeanin A, a natural oleanane-type triterpenoid, has been shown to exert pro-apoptotic effects in colorectal cancer cell lines (SW480 and LOVO) through inhibition of Wnt/β-catenin signaling. Treatment with Raddeanin A reduced β-catenin levels in both the cytoplasm and nucleus while increasing the levels of cytoplasmic phosphorylated β-catenin. Further analysis revealed that Raddeanin A also targets additional components of the Wnt signaling cascade, including LRP6, PI3K/AKT, and GSK-3β [134].

### 4.9. The Hippo Signaling Pathway

**Figure 14:**
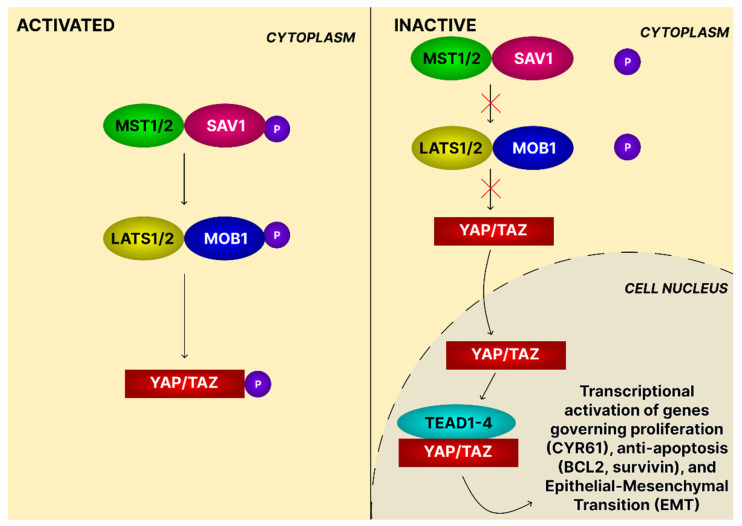
Simplified diagram of the Hippo pathway. Phosphorylation of LATS1/2, thanks to the activity of MST1/2 and SAV1, leads to phosphorylation of YAP/TAZ, which retains this complex in the cytoplasm. Inhibition of Hippo signaling leads to translocation of unphosphorylated YAP/TAZ to the nucleus, binding TEADs, and initiating the expression of genes regulating proliferation, apoptosis, and EMT. The arrow indicates a stimulating effect. The crossed arrows indicate an inhibition.

The Hippo, also known as the Salvador/Warts/Hippo (SWH) pathway, plays a fundamental role in tissue homeostasis and cellular regulation. Hippo pathway activation is modulated by diverse intracellular and extracellular cues, including cell–cell contact, ECM composition, mechanical stress, energy status, and cellular stress. Mechanistically, Mammalian Ste20-like serine/threonine (MST1/2) kinases, together with Salvador homolog 1 (SAV1), phosphorylate and activate Large tumor suppressor 1/2 (LATS1/2), and then phosphorylate YAP/TAZ, retaining them in the cytoplasm and preventing transcriptional activity. When Hippo signaling is suppressed, unphosphorylated YAP/TAZ translocate to the nucleus, bind TEA domain (TEAD) transcription factors, and drive expression of genes regulating proliferation, survival, and apoptosis (Figure 14) [135].

The Hippo pathway is among the most frequently dysregulated signaling cascades in human malignancies. YAP and TAZ act as potent oncogenic drivers, whereas MST1/2 and LATS1/2 function as tumor suppressors. Hippo inactivation enhances proliferation, inhibits apoptosis and contributes to tumor initiation. Moreover, Hippo signaling regulates cancer metabolism, including glycolysis, supplying energy and biosynthetic substrates for tumor growth. CSCs, critical for tumor initiation, are regulated by TAZ; depletion of TAZ diminishes tumor-seeding potential. Interestingly, the pathway exhibits tissue-specific duality, acting as a tumor suppressor in certain contexts: YAP overexpression can inhibit colorectal tumor growth and metastasis, and similar suppressive effects are reported in estrogen receptor (ER)-positive breast cancer, hematologic malignancies, and neural/neuroendocrine tumors. LATS1/2 deletion in immunocompetent mouse models paradoxically enhances anti-tumor immunity, highlighting context-dependent outcomes [136,137,138].

Genomic and transcriptomic analyses show that Hippo pathway alterations, particularly involving YAP1 and MST1, are strongly linked to high-grade serous ovarian carcinoma (HGSOC) and serve as diagnostic and prognostic markers. Frequent amplifications of Hippo genes in advanced HGSOC enhance YAP nuclear activation, enhancing chemoresistance driving proliferation and survival, while high Hippo gene expression correlates with poorer overall survival [20].

Xia et al. [139] evaluated the effect of YAP, a key component of the Hippo signaling pathway, on ovarian cancer progression. Researchers observed that hyperactivation of YAP (resulting from the introduced YAP-5SA mutation) increased proliferative activity, chemoresistance, migration, and invasiveness of tumor cells, which correlated with poor prognosis for patients.

Kim et al. [140] examined the effect of ursolic acid on the activation of Hippo pathway components in SNU484 and SNU638 gastric cancer cells. Ursolic acid treatment resulted in a decreased viability, increased proapoptotic activity, and reduced invasiveness and migration of cancer cells. Triterpene enhanced the expression of Ras association domain family protein 1 (RASSF1), MST1/2, and LATS. In turn, the expression of YAP1, forhead box M1 (FOXM1), KRAS, and basic leucine zipper transcription factor (BATF) was declined. In the case of YAP, its phosphorylated level increased, leading to the formation of an inactive form and, consequently, activation of the Hippo signaling pathway in cancer cells. Moreover, RASSF1 knockdown led to significant inhibition of p-YAP expression, which was further reduced when UA was added. In vivo, Hippo pathway protein levels were also increased compared to controls.

Interactions between the Hippo/YAP and PI3K/AKT/mTOR pathways may represent new therapeutic targets. Effector components of the Hippo pathway, i.e., YAP and TAZ proteins, may increase the expression of PI3K/AKT pathway components, which translates into increased proliferative activity of cells and cancer progression. The relationship between the Hippo/YAP and PI3K/AKT pathways has been observed in endometrial, oral, liver, colon, and hepatocellular carcinomas. Therefore, activation of YAP1 is tightly correlated with poor differentiation, advanced stage, and poorer survival in these tumors [136]. Meng et al. [141] evaluated the effect of ursolic acid combined with 3,3′-diindoylmethane (DIM) on the Hippo/YAP and AKT pathways in esophageal cancer cells. The study found that this combination inhibited the AKT/Gsk-3β signaling pathway and activated the Hippo tumor suppressor pathway. Furthermore, the use of the AKT inhibitor LY294002 and the PI3K inhibitor 3-methyladenine intensified the inhibitory effect of the tested complex on AKT pathway activation and YAP nuclear translocation in cells. YAP suppression increased the activation of the tumor suppressor PTEN, which in turn reduced p-AKT expression. In vivo results confirmed that combined treatment (UA with DIM) effectively reduced esophageal tumors via feedback between the PI3K/AKT and Hippo pathways.

Another combination therapy is the combination of ursolic acid with a well-known chemotherapeutic agent such as doxorubicin. Hu et al. [142] examined the effect of this combination on the AKT and Hippo signaling pathways in colon cancer cells. Their findings demonstrated that UA enhanced the anti-cancer activity of doxorubicin. In vitro and in vivo (mouse model) studies led to the conclusion that this effect was due to blockade of the AKT/Gsk3β kinase signaling pathway and activation of the Hippo tumor suppressor pathway, which reduced the expression of the YAP effector protein and connective tissue growth factor (CTGF) in colon cancer cells. The additional introduction of the PI3K inhibitor, LY294002, increased the inhibition not only of AKT activity but also of the activity of components involved in the Hippo tumor suppressor pathway in cells incubated with the tested compounds. This resulted in inhibition of Yap and CTGF expression. Introduction of the AKT activator, SC79, resulted in the induction of CTGF expression. Based on the obtained in vitro and in vivo results, it was concluded that co-administration of ursolic acid with doxorubicin significantly inhibited colon cancer progression.

### 4.10. The Hedgehog Signaling Pathway

**Figure 15:**
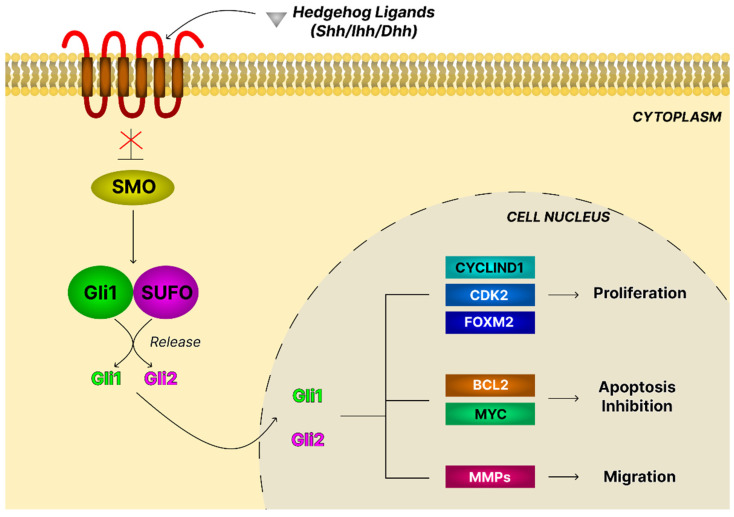
Simplified diagram of the Hh pathway. Hedgehog ligands binding relieves inhibition, enabling Smo activation and subsequent regulation of Gli transcription factors. Activated Gli proteins translocate to the nucleus and induce target gene expression controlling cell proliferation, differentiation, and survival. The arrow and line indicate a stimulating effect. The crossed line ending with perpendicular bar indicates the unblocking of inhibition.

The Hh pathway is a highly conserved regulatory system essential for embryonic development, tissue homeostasis, and regeneration. In mammals, it comprises three ligands, Sonic hedgehog (Shh), Indian hedgehog (Ihh), and Desert hedgehog (Dhh), which evolved from a single Hh ligand in Drosophila. Pathway activation occurs when Hh ligands bind the transmembrane receptor Patched (Ptch1), relieving its inhibition of the G-protein-coupled receptor Smoothened (Smo) and triggering Gli transcription factor activation. Gli proteins translocate to the nucleus to regulate genes controlling proliferation, differentiation, and survival (Figure 15) [143].

Inhibition of Hh sensitizes tumors to TRAIL-induced apoptosis, disrupts anti-apoptotic feedforward loops, and modulates autophagic flux. Hh also contributes to chemoresistance and radioresistance via TGF-β and TNF-α induction, mTOR/S6K1 activation, and atypical kinase-mediated Gli feedback, promoting tumor repopulation after therapy [144].

Aberrant Hh/Gli activation is central to ovarian cancer pathogenesis. Gene expression and cytogenetic analyses reveal frequent overexpression of Shh ligands, Ptch, Smo, and Gli1/Gli2 in ovarian tumors. Elevated Gli1 correlates with advanced disease and reduced patient survival. Moreover, Hh activation correlates with chemoresistance, as hyperactive cells exhibit enhanced survival and DNA repair, whereas pathway inhibition sensitizes tumors to therapy and reduces proliferation [21].

Recent findings indicate that betulinic acid, a pentacyclic triterpenoid, can modulate Hedgehog signaling by targeting its key downstream effectors. Wang et al. [67] demonstrated that treatment of RMS-13 human ovarian cancer cells with a combination of BA and 5-fluorouracil for 48 h significantly reduced the mRNA expression of Gli1, Gli2, Ptch1, and IGF-2. Interestingly, the inhibitory action of the betulinic acid and 5-fluorouracil combination on Gli1 activity was independent of cyclopamine, a specific inhibitor of SMO, indicating that betulinic acid acts downstream of SMO, likely at the level of GLI transcription factors. Supporting this hypothesis, in NIH-3T3 cells transiently activated with Sonic Hedgehog, the combination treatment selectively suppressed Gli1 and Gli2 expression, while leaving Ptch1 and IGF-2 levels largely unaffected. Taken together, these findings indicated that the combination of 5-fluorouracil and BA selectively inhibited Hedgehog signaling through Gli family transcription factors.

## 5. Conclusions

Pentacyclic triterpenoids are a widely occurring group of plant-derived compounds with a broad spectrum of biological activities. To utilize them in effective and safe therapy, a detailed understanding of the molecular mechanisms underlying their action on disease-specific signaling pathways is essential. During carcinogenesis, alterations in numerous molecular pathways accumulate, contributing to tumor progression and reducing the patient’s chances of recovery. For these reasons, it seems reasonable to precisely control the induction or suppression of specific signaling pathways to improve patient prognosis.

This article presents the current status of research on the effects of PTs and their derivatives on the PI3K/AKT/mTOR, MAPK/ERK, NF-κB, JAK/STAT, Notch, HIF-1α, TGF-β, Wnt/β-catenin, Hippo, and Hedgehog pathways, which are involved in regulating, e.g., proliferation, epithelial-to-mesenchymal transition, autophagy, and apoptosis in cancer cells (Table 1). Some pathways are more frequently modulated by triterpenoid-based compounds, for example, PI3K/AKT/mTOR or MAPK/ERK, than others, such as HIF-1α or Hedgehog, which is also reflected in the amount of available literature. This warrants increased research attention in the near future.

Among the PTs and their derivatives, it is possible to identify compounds that negatively regulate one (e.g., bartogenic acid, alpha-hederin, β-escin, asiatic acid, β-boswellic acid, uvaol, betulin, taraxerol) or several (e.g., oleanolic acid, ursolic acid, betulinic acid, celastrol, pristimerin) of the above-mentioned pathways simultaneously. Based on the presented literature, it can be concluded that unmodified ursolic acid exhibits the most diverse activity in terms of modulating signaling pathways (PI3K/AKT/mTOR, MAPK/ERK, JAK/STAT, HIF-1α, TGF-β, Wnt/β-catenin, and Hippo). Regardless of the cancer cell line, signaling modulation using this triterpenoid most often included suppression of cell growth, induction of pro-apoptotic processes, and inhibition of cell migration and invasiveness. Similar biological results were observed for the other strong modulator of signaling pathways, pure oleanolic acid.

When considering the therapeutic potential of pentacyclic triterpenoids, it is important to remember that they exhibit limited water solubility, low intestinal absorption, rapid metabolism, and nonspecific distribution. Therefore, new strategies are being explored to improve their physicochemical properties, bioavailability, and consequently expand possibilities for their effective use in anti-cancer therapy [145]. One of the most fundamental approaches is direct modification of the chemical structure of a selected compound. For example, betulin contains three positions in the side chain, C-3, C-19, and C-28, that can be relatively easily modified. It should be noted that structural changes may influence not only physicochemical properties but also biological activity. In a study by Chodurek et al. [60], the anti-cancer activity of betulin and its propynoyl derivatives was evaluated in vivo in SK-OV-3 and OVCAR-3 ovarian cancer cells. The chemical modifications of betulin at the C-28 and C-3 positions with propynoyl groups, and at the C-3, C-28, and C-30 positions with phosphate or C-29 phosphonate groups, allow the formation of compounds with greater cytotoxic activity than the parent molecule that could be used in development of the new pharmaceutical dosage forms for the treatment of ovarian cancer.

Another lupane-type pentacyclic triterpenoid, betulinic acid, like betulin, exhibits strong inhibitory effects on cell growth, promotes apoptosis, and limits cell migration. To improve its inherent properties, not the direct chemical modifications but numerous nanotechnology-based strategies have been developed to enable controlled, targeted, and stimulus-responsive release of the active substance at the tumor site, improving therapeutic efficacy and reducing toxicity to healthy cells. BA has been incorporated into various nanocarriers, e.g., nanoparticles, nanogels, liposomes, micelles, and nanosuspensions, which improve its solubility, stability, bioavailability, anti-tumor activity, and reduce chemotherapy-related side effects. With continued technological advances, BA-based nanomedicines hold strong potential for future clinical application, offering safer and more effective options for cancer treatment than standard solutions [146].

Not only lupane-type derivatives but also other pentacyclic triterpenoids can be chemically modified to improve their physicochemical and biological properties, as demonstrated in this study. The synthetic derivatives based on oleanolic acid such as CDDO-Me [100] and CDDO-Im [118] modulate signaling pathways, including NF-κB, MAPK/ERK, Notch, and TGF-β, thereby limiting cell proliferation, triggering apoptotic processes, and inhibiting EMT and cell invasiveness that significantly impacts the survival chances of oncology patients.

Terpenoids may be administered alone or in combination with standard chemotherapeutics. Such combination therapy enables synergistic effects and often significantly reduces the risk of development of multidrug resistance. This paper presents selected examples of combination therapies, in which ursolic acid enhances the effects of 3,3′-diindolylmethane [141] or doxorubicin [142], while betulinic acid enhances the activity of 5-fluorouracil [67].

As previously mentioned, in the case of betulinic acid, specific nanoformulations with pentacyclic triterpenoids can be developed, aiming to improve their pharmacokinetics, biodistribution, and therapeutic efficiency. Such nanoformulations enable enhanced cellular uptake, promote tailored release profiles, and increase the cytotoxic effect of the compounds on cancer cells [147].

One of the most innovative approaches in the context of modern nanoformulations is the use of self-assembling systems. They can overcome the drawbacks characteristic of standard carriers used for encapsulation of actives, such as low loading capacity, insufficient biocompatibility, the risk of immunogenicity, and local or systemic toxicity. Interestingly, certain pentacyclic triterpenoids can form such self-assembling, biocompatible, biodegradable, and nontoxic carriers. For example, extended-release nanoparticles made of oleanolic acid with paclitaxel exerted a significantly stronger anti-cancer effect than paclitaxel alone while markedly reducing hepatotoxicity. The results confirmed the possibility of achieving synergistic effects while minimizing adverse effects [145]. Nanoparticles made from ursolic acid can also deliver paclitaxel. This system showed prolonged circulation time, significantly enhanced tumor-growth inhibition (3.3-fold greater than paclitaxel alone) and markedly reduced liver toxicity [148]. A highly needed solution is the development of nanoparticles capable of crossing the blood–brain barrier, which may be used to treat lesions that are difficult to reach with conventional therapies. In a study by Deng et al. [149], it was shown that betulinic acid and glyburide could be used together in combination therapy in ischemic stroke treatment and reducing the risk of hypoglycemia in the circulatory system.

More complex systems, called multi-component co-assemblies, can also be created, for example, a combination of oleanolic acid, glycyrrhetinic acid, and paclitaxel. Wang et al. [150] determined the tumor inhibition rate was 82.6 to 23.7% higher than paclitaxel alone. The last assembled strategy involves carrier-free nanodelivery systems, in which the core is made of a triterpenoid, like ursolic acid, and the shell consists of epigallocatechin gallate. These systems are pH-responsive, meaning that active substances are released in acidic environments typical of inflammatory and cancerous lesions. As a result, they provide effective tumor-growth inhibition and apoptosis induction, increasing therapeutic efficacy while improving safety levels [145].

The presented collective findings on the use of pentacyclic triterpenoid-based compounds as modulators of cell survival and proliferation mechanisms in cancer cell models clearly indicate, firstly, the need for further research on their molecular targets and biological effects, especially regarding the less well-known signaling interactions, and secondly, the importance of focusing on the development of advanced formulations that enhance the efficacy of potential therapies while maintaining a high safety profile for oncology patients.

## Figures and Tables

**Table 1:** Summary table integrating the data about selected pentacyclic triterpenoids and their derivatives, taking into account their molecular targets and observed biological effects in various cancer cell models. ↑ indicates an induction; ↓ indicates an inhibition.

Type of Structure	Compound	Molecular Target	Biological Effect	Cancer Cell Model	Reference
Oleanane-type	Oleanolic acid	↓: AKT/mTOR/S6K	↑: autophagy; ↓: cell proliferation, invasiveness	KRAS-transformed MCF10A xenografted mouse	[76]
		↓: MAPK/ERK	↓: migration, invasiveness	U-87MG, U-251MG	[87]
		↓: MAPK/ERK	↑: apoptosis; ↓: cell proliferation	DU145	[91]
		↑: MAPK/ERK	↑: apoptosis; ↓: cell proliferation	MCF-7, U87	[91]
		↑: MAPK/ERK	↑: apoptosis; ↓: cell proliferation	HepG2	[92]
	Bartogenic acid	↓: NF-κB	↓: cell viability, necrosis	SK-OV-3 xenografted mouse	[46]
	Glycyrrhetinic acid	↓: MAPK/ERK	↑: apoptosis; ↓: cell proliferation, angiogenesis	A2780, HUVEC	[48]
	Glycyrrhizic acid	↑: p21/WAF1; ↓: Notch	↓: cell proliferation	HPV16+ CaSki	[116]
	Alpha-hederin	↓: PI3K/AKT/mTOR	↑: apoptosis	SK-OV-3, SCC-25	[79,80]
	Eclalbosaponin II	↑: MAPK; ↓: PI3K/AKT/mTOR	↑: apoptosis, autophagy; ↓: cell proliferation	SK-OV-3, A2780	[81]
	β-escin	↓: NF-κB	↓: migration, invasiveness	B16F10, SK-MEL5	[103]
	Theasaponin E1	↓: PI3K/AKT/mTOR, Notch, HIF-1α	↑: apoptosis; ↓: cell proliferation, migration, angiogenesis	OVCAR-3	[123]
	Raddeanin A	↓: PI3K/AKT/mTOR, Wnt/β-catenin, NF-κB	↑: apoptosis; ↓: cell proliferation	SW480, LOVO	[134]
	CDDO-Me	↓: NF-κB	↑: apoptosis, oxidative stress	OVCAR-5, MDAH-2774	[100]
	CDDO-Im	↓: Notch, TGF-β	↑: apoptosis; ↓: cell proliferation, EMT	SUM159	[118]
Ursane-type	Ursolic acid	↓: PI3K/AKT/mTOR	↑: apoptosis; ↓: autophagy	SK-OV-3	[77]
		↓: MAPK/ERK	↑: apoptosis; ↓: cell proliferation	CAOV3	[84]
		↑: MAPK/ERK	↑: apoptosis; ↓: cell proliferation	SK-OV-3	[93]
		↓: JAK/STAT3	↑: antioxidative, anti-ferroptosis	NRK-52E	[107]
		↓: STAT3	↓: cell viability	A549	[109]
		↓: JAK/STAT3	↑: apoptosis; ↓: cell proliferation, colony formation, angiogenesis, migration, invasiveness	A549, H460	[110]
		↓: STAT3	↑: apoptosis	HCT116, HT29	[111]
		↓: PI3K/AKT/mTOR, HIF-1α	↓: adaptation to hypoxic stress, angiogenesis	SK-OV-3	[122]
		↓: TGF-β1	↓: cell proliferation, migration, invasiveness	HCT-116, HCT-8	[130]
		↑: p53; ↓: Wnt/β-catenin	↓: cell proliferation	143B	[132]
		↓: PI3K/AKT/mTOR, Wnt/β-catenin	↑: apoptosis; ↓: cell proliferation, migration, clonality	SW620	[133]
		↑: Hippo	↑: apoptosis; ↓: cell viability, migration, invasiveness	SNU484, SNU638	[140]
	2α,3β-dihydroxy-urs-12-en-28-oic acid	↓: NF-κB	↑: apoptosis	BGC823	[106]
	Ursolic acid-derivatives with a diamide gallic acid moiety	↓: NF-κB	↑: apoptosis; ↓: cell proliferation, migration	SK-OV-3, A549, HepG2, T24	[101]
	Ursolic acid with 3,3′-diindoylmethane	↑: Hippo; ↓: PI3K/AKT/mTOR	↑: apoptosis; ↓: cell proliferation, migration	TE-8, TE-12	[141]
	Ursolic acid with doxorubicin	↑: Hippo; ↓: PI3K/AKT/mTOR	↑: apoptosis; ↓: cell proliferation, migration, EMT	HCT116, HT29	[142]
	Asiatic acid	↓: PI3K/AKT/mTOR	↑: apoptosis; ↓: cell viability, colony formation	OVCAR-3, SK-OV-3	[55]
		↑: MAPK/ERK	↑: apoptosis; ↓: cell proliferation	MCF-7, MDA-MB-231	[94]
		↓: Notch	↓: inflammation, mitochondrial dysfunction	RAW264.7	[117]
		↓: TGF-β1	↓: EMT	SK-OV-3	[58]
		↓: TGF-β1	↓: EMT	A549	[128]
	β-Boswellic acid	↓: NF-κB	↑: apoptosis; ↓: cell proliferation	PC-3	[104]
	Uvaol	↓: TGF-β1	↓: EMT	A549	[129]
Lupane-type	Lupeol	↓: MAPK/ERK	↑: apoptosis; ↓: cell proliferation, migration	UPCI:SCC154, UPCI:SCC090	[85]
	Betulin	↓: PI3K/AKT/mTOR	↑: apoptosis; ↓: cell proliferation, migration, invasiveness	OVCAR-3	[61]
	Betulinic acid	↓: MAPK/ERK	↑: autophagy; ↓: cell proliferation, migration, invasiveness	SGC-7901	[86]
		↑: p21/WAF1; ↓: NF-κB	↑: apoptosis	LNCaP, DU145	[105]
		↓: JAK/STAT3	↑: apoptosis	U266	[113]
		↓: STAT3	↑: apoptosis	A293	[113]
	Betulinic acid with 5-fluorouracil	↓: Hedgehog	↑: apoptosis; ↓: cell proliferation	OVCAR 432, RMS-13	[67]
	3-O-(E)-p-coumaroylbetulinic acid	↓: Notch	↑: apoptosis; ↓: cell viability, cell proliferation	MBA-MD-231, T47D	[119]
Friedelane-type	Celastrol	↓: PI3KAKT/mTOR, NF-κB	↑: apoptosis; ↓: cell proliferation	OVCAR-3, BGC823, MGC803	[70,82]
		↓: PI3KAKT/mTOR	↑: apoptosis	B16	[83]
		↓: NF-κB	↓: migration, invasiveness	OVCAR-3, SK-OV-3	[102]
		↓: STAT3	↓: fibrosis, hypertrophy	rat cardiomiocytes	[112]
	Pristimerin	↓: PI3KAKT/mTOR, NF-κB	↑: apoptosis; ↓: cell proliferation	OVCAR-5, MDAH-2774, OVCAR-3, SK-OV-3	[69]
Taraxastane-type	Taraxerol	↓: PI3K/AKT/mTOR	↑: apoptosis	LPS-induced RAW264.7, HeLa	[78]
Multiflorane-type	Multiflorane	↓: MAPK/ERK	↑: apoptosis ↓: cell proliferation, migration, invasiveness	U87	[90]

## Data Availability

No new data were created or analyzed in this study. Data sharing is not applicable to this article.

## Abbreviations

The following abbreviations are used in this manuscript:

AA	Asiatic acid
ABCG2	Adenosine triphosphate (ATP)-binding cassette efflux transporter G2
APC	Adenomatous polyposis coli
ARNT	Aryl hydrocarbon receptor nuclear translocator
ATM	Ataxia telangiectasia mutated
β-TRCP	β-transducin repeat-containing protein
BA	Betulinic acid
BAD	Bcl-2-associated agonist of cell death
BATF	Basic leucine zipper transcription factor
Bax	Bcl-2 associated X
Bcl-2	B-cell CLL/lymphoma 2
Bcl-xL	B-cell lymphoma-extra large
BM (CDDO-Me)	Bardoxolone methyl
BMPs	Bone morphogenetic proteins
BRCA	Breast Cancer Gene
BTK	Bruton tyrosine kinase
CDDO-Im	2-cyano-3,12-dioxooleana-1,9(11)-dien-28-oic acid imidazolide
CDK	Cyclin-dependent kinase
CDKN2A/B	Cyclin-dependent kinase inhibitor 2A/B
cIAP	Inhibitor of apoptosis
CK1α	Casein kinase 1α
COX	Cyclooxygenase
CRA	2α,3β-dihydroxy-urs-12-en-28-oic acid
CSCs	Cancer stem cells
CSL	Supressor of Hairless
CTGF	Connective tissue growth factor
Dhh	Desert hedgehog
DIM	3,3′-diindoylmethane
DLL	Delta-like
DUSPs	Dual-specificity phosphatases
Dvl	Dishevelled
ECM	Extracellular matrix
EGFR	Epidermal growth factor receptor
EMT	Epithelial–mesenchymal transition
EPHA5	Ephrin type-A receptor 5
EPO	Erythropoietin
ER	Estrogen receptor
ERK	Extracellular signal-regulated kinase
FCGBP	Fc gamma binging protein
FOXO	Forkhead box O
Fzd	Frizzled (Fzd)
FOXM1	Forhead box M1
GA	Glycyrrhetinic acid
GARP	Glycoprotein A repetitions predominant
GPCRs	G protein-coupled receptors
GSK3β	Glycogen synthase kinase-3 beta
HER2	Human epidermal growth factor receptor 2
HGSOC	High-grade serous ovarian carcinoma
Hh	Hedgehog
HIFs	Hypoxia-inducible factors
HREs	Hypoxia-response elements
HUVECs	Human umbilical vein endothelial cells
IC_50_	Half maximal inhibitory concentration
IGF-1	Insulin-like growth factor 1
Ihh	Indian hedgehog
IKK	IkappaB kinase
IL	Interleukin
INF	Interferon
KRAS	Kristen rat sarcoma viral oncogene homolog
JAK	Janus kinase
LAP	Latency-associated peptide
LATS1/2	Large tumor suppressor 1/2
LC3	Microtubule-associated protein 1A/1B-light chain
LLCs	Large latent complexes
LOX	Lipooxygenase
LRP5/6	Low-density lipoprotein receptor-related proteins 5 and 6
LTBP	Latent transforming growth factor beta binding protein
MAML	Mastermind-like
Mcl-1	Myeloid leukemia-1
MDM2	Murine double minute 2
MEK	Mitogen-activated protein kinase
MMP	Metaloproteinase
MST1/2	Mammalian Ste20-like serine/threonine kinases
mTORC	Mechanistic target of rapamycin complex
NECD	Extracellular domain
NEMO	NF-κB essential modulator
NFAT	Nuclear factor of activated T-cells
NF-κB	Nuclear factor kappa-light-chain-enhancer of activated B cells
NICD	Intracellular domain
NIK	NF-κB-inducing kinase
OA	Oleanolic acid
OC	Ovarian cancer
PAMPs	Pathogen-associated molecular patterns
PCP	Wnt/Planar Cell Polarity
PDK1	Phosphoinositide-dependent protein kinase-1
PD-L1	Programmed death-ligand 1
PHDs	Oxygen- and iron-dependent prolyl hydroxylases
PI3K/AKT/mTOR	Phosphatidylinositol-3-kinase/AKT/mammalian target of rapamycin
PIAS	Protein inhibitors of activated STATs
PIK3CA	Phosphatidylinositol-4,5-bisphosphate 3-kinase catalytic subunit alpha
PIP2	Phosphatidylinositol-4,5-bisphosphate
PIP3	Phosphatidylinositol-3,4,5-trisphosphate
PORCN	Porcupine
Ptch1	Transmembrane receptor Patched
PTEN	Phosphate and tensin homolog
PTPs	Protein tyrosine phosphatases
PTs	Pentacyclic triterpenoids
pVHL	von Hippel–Lindau ubiquitin ligase complex
Raf	Rapidly accelerated fibrosarcoma
Ras/MAPK	Rat sarcoma virus/mitogen-activated protein kinase
RASSF1	Ras association domain family protein 1
RGD	Arg-Gly-Asp
ROCK	Rho-associated protein kinase
ROS	Reactive oxygen species
RTKs	Receptor tyrosine kinases
S6K	Ribosomal protein S6 kinase
SAV1	Salvador homolog 1
SH2	Src homology 2
Shh	Sonic hedgehog
SIRT1	Sirtuin1
Smac	Second mitochondrial-derived activator of caspase
Smo	G-protein-coupled receptor Smoothened
SOCS	Suppressors of cytokine signaling
SREBP1	Sterol regulatory element-binding protein 1
STAT3	Signal transducer and activator of transcription 3
SWH	Salvador/Warts/Hippo pathway
TβRI, TβRII	TGF-β type I and type II receptors
TCF/LEF	T-cell factor/lymphoid enhancer factor
TCTP	Translationally controlled tumor protein
TEAD	TEA domain
TGF-β	Transforming growth factor
TIMP	Tissue inhibitors of metalloproteinase
TLE	Transducin-like enhancer of split
TMD	Transmembrane region
TNBC	Triple-negative breast cancer
TNF-α	Tumor necrosis factor-α
TRAIL-APO-2L	TNF-related apoptosis-inducing ligand/apoptosis ligand 2
TSP-1	Thrombospondin-1
UA	Ursolic acid
U-STATs	Unphosphorylated STATs
VEGF	Vascular endothelial growth factor
VEGFR-2	Vascular endothelial growth factor receptor-2 (VEGFR-2)
YAP/TAZ	Yes-associated protein/transcriptional coactivator with PDZ-binding motif
ZEB1	Zinc finger E-box binding homeobox 1
